# Aphid parasitism alters induced plant responses allowing a hyperparasitoid to locate its hidden parasitoid host

**DOI:** 10.1111/nph.70774

**Published:** 2025-12-15

**Authors:** Mitchel E. Bourne, Alessia Vitiello, Gabriel A. Charvalakis, Leandra Meerkerk, Berhane T. Weldegergis, Karen J. Kloth, Erik H. Poelman

**Affiliations:** ^1^ Laboratory of Entomology Wageningen University & Research Droevendaalsesteeg 1 6708 PB Wageningen the Netherlands; ^2^ National Centre for Vector Entomology, Institute of Parasitology, Vetsuisse and Medical Faculty, One Health Institute University of Zürich Winterthurerstrasse 266A 8057 Zürich Switzerland

**Keywords:** *Alloxysta fuscicornis*, EPG, host‐location, multitrophic interactions, plant transcriptome, plant‐mediated interactions

## Abstract

- Plants typically host insect communities composed of multiple trophic levels that are intricately linked through interactions mediated by the shared food plant. Hyperparasitoids are top‐level carnivores in such systems, preying on parasitoid larvae developing inside herbivores. These hyperparasitoids can dramatically reduce the effectiveness of aphid biological control, but how they locate parasitised aphids remains unknown.
- We compared two aphid–parasitoid systems occurring on the same host plant to test whether a common aphid hyperparasitoid uses plant volatiles induced by parasitised aphids to locate its host. We combined behavioural assays with transcriptomic analyses, volatile profiling and aphid feeding behaviour measurements to investigate the underlying mechanisms.
- The aphid hyperparasitoid responded to volatile cues induced specifically by its primary host–parasitoid association to locate its concealed host. Transcriptomic analysis indicated that parasitism attenuated plant defence responses to aphid feeding, with changes in homoterpene biosynthesis observed only in the primary host–parasitoid association. In both systems, parasitised aphids increased their xylem feeding activity.
- These findings show that parasitism reshapes plant signalling in ways that enable hyperparasitoids to detect hidden hosts via herbivore‐induced cues. This interaction web mirrors caterpillar‐based systems, despite fundamental differences in herbivore feeding strategies and their induced plant responses.

## Samenvatting

- Planten huisvesten insecten afkomstig van meerdere trofische niveaus die nauw met elkaar verbonden zijn door interacties met de gedeelde (voedsel)plant. Hyperparasitoïden zijn top carnivoren in zulke systemen, ze jagen op larven van sluipwespen (parasitoïden) die zich in herbivore insecten ontwikkelen. Hierdoor kunnen hyperparasitoïden de effectiviteit van biologische bestrijding van bladluizen drastisch verminderen, maar hoe ze geparasiteerde bladluizen lokaliseren, is nog onbekend.
- We vergeleken twee bladluis‐sluipwesp associaties die op dezelfde waardplant voorkomen, om te testen of een veelvoorkomende bladluis‐hyperparasitoïde plantengeuren gebruikt om zijn gastheer te lokaliseren. Hiervoor combineerden we gedrags‐experimenten met transcriptomische analyses, analyses van plantengeuren en het voedingsgedrag van bladluizen om ook de onderliggende mechanismes te onderzoeken.
- De bladluis‐hyperparasitoïde reageerde op de plantengeuren die werden geïnduceerd door de meest voorkomende bladluis‐sluipwesp associatie om haar verborgen gastheer te lokaliseren. Transcriptomische analyse gaf aan dat parasitisme de afweerreacties van planten op bladluizen verzwakte, waarbij alleen in de meest voorkomende bladluis‐sluipwesp associatie veranderingen in homoterpeen‐biosynthese werden waargenomen. In beide systemen hadden geparasiteerde bladluizen een verhoogde xyleemvoedingsactiviteit.
- Deze bevindingen tonen aan dat parasitisme de signaaloverdracht van planten zodanig verandert dat hyperparasitoïden hun verborgen gastheren kunnen detecteren. Dit interactienetwerk lijkt sterk op bekende processen bij rupsen, ondanks fundamentele verschillen in de voedingsstrategieën tussen rupsen en bladluizen, en de door hen geïnduceerde reacties van planten.

## Introduction

Trophic interactions within food webs, such as consumer‐resource, predator–prey and host–parasite relationships form the backbone of ecological communities (Price *et al*., 1980; Dicke & Grostal, 2001). These direct trophic chains are linked through indirect interactions that arise from feedback loops on species abundance and plasticity in trait expression by species interactions (Fox & Potts, 2022). In terrestrial food webs, plant phenotypic responses to herbivory result in indirect plant‐mediated interactions impacting a wide range of community members such as other herbivores, pathogens, predators and pollinators (Ohgushi, 2005; Utsumi *et al*., 2010; Barbour *et al*., 2022). Such induced plant responses therefore provide a mechanistic basis for the organisation of complex multitrophic food webs.

Endoparasitoid wasps, which lay their eggs inside the bodies of other insects, are a striking example of how indirect interactions can cascade through food webs (Poelman & Cusumano, 2022). By modifying herbivore physiology and their secretions, parasitism can affect plant defence responses to herbivores (Zhu *et al*., 2015; Tan *et al*., 2018) and subsequently herbivore performance (Poelman *et al*., 2011a; Cuny *et al*., 2022). In addition, herbivore‐induced plant volatile (HIPV) emissions are altered when plants are damaged by parasitised herbivores (Zhu *et al*., 2015). Such parasitism‐altered HIPVs affect information transfer towards other interactors in the food web (Poelman *et al*., 2012), consequently affecting herbivore preference (Cusumano *et al*., 2018), deterring other parasitoids from the plant preventing superparasitism (Fatouros *et al*., 2005; Kafle *et al*., 2020) and even extending to interactions across four trophic levels by attracting the natural enemies of parasitoids, the so‐called hyperparasitoids (Poelman *et al*., 2012).

Hyperparasitoids play a key role in regulating the populations of parasitoids and herbivores and thus have a significant impact on the structure and dynamics of food webs (Rosenheim, 1998; Frago, 2016). They are thought to enforce the strength of interactions in terrestrial food webs as their presence prevents large oscillations in predator–prey‐like interactions (Rosenheim, 1998; Tougeron & Tena, 2019). However, in crop systems, hyperparasitoids are considered pests, as they reduce the efficacy of biological control strategies involving parasitoids (Tougeron & Tena, 2019; Cusumano *et al*., 2020). Aphid hyperparasitoids are especially problematic as their presence can lead to a complete collapse of parasitoid populations (de Boer *et al*., 2019; Tougeron & Tena, 2019). Yet aphid hyperparasitoids are understudied and their host‐location strategy is largely unknown.

Previous research failed to pinpoint whether aphid hyperparasitoids use HIPVs to locate their host (Buitenhuis *et al*., 2005; de Boer *et al*., 2020). To date, this phenomenon has only been demonstrated for hyperparasitoids in Lepidopteran caterpillar‐based systems. The altered plant phenotype that attracts hyperparasitoids has been causally linked to a symbiotic polydnavirus transferred from the parasitoid wasp to a caterpillar, which modifies the caterpillar's saliva and regurgitate and thereby changes plant responses (Cusumano *et al*., 2018, 2025; Tan *et al*., 2018). Whether similar mechanisms exist in aphid systems is especially intriguing because aphids differ markedly from caterpillars in feeding mode, the plant responses they elicit and aphid parasitoids do not carry a symbiotic polydnavirus. Aphid feeding is known to prominently activate salicylic acid (SA)‐ and ethylene (ET)‐mediated defences rather than the jasmonic acid (JA)‐dominated defences activated by caterpillars (Pieterse *et al*., 2012; Aerts *et al*., 2021). Yet parasitised aphids also show phenotypic similarities to parasitised caterpillars. For example, plants infested with parasitised aphids induce an altered expression of a defence‐related gene (Vaello *et al*., 2018), mirroring responses to parasitised caterpillars (Poelman *et al*., 2011b; Tan *et al*., 2018). Moreover, parasitised aphids remain alive and interact continuously with the plant for several days, similar to parasitised caterpillars that continue to feed and provide cues for hyperparasitoids.

Previous studies on aphid hyperparasitoids have mainly focussed on pseudo‐hyperparasitoids, which attack cocoons of parasitoids that are formed when parasitoid larvae have pupated (so‐called mummies) (Poelman *et al*., 2022). Due to their generalist nature and requirement to find parasitoid larvae after aphids die and no longer interact with the plant, their host location is likely not guided by HIPVs (Buitenhuis *et al*., 2005; de Boer *et al*., 2020; Poelman *et al*., 2022). True aphid hyperparasitoids lay eggs inside the larvae of their parasitoid host. Therefore, true aphid hyperparasitoids must find newly parasitised aphids before they mummify, giving them a limited time frame to locate their host (Sullivan & Völkl, 1999; Poelman *et al*., 2022). In addition, they are known to have a higher degree of specialisation (Grasswitz, 1998; Brodeur, 2000), and their parasitised aphid host still interacts with the plant, making it more likely that they are sensitive to subtle changes in plant phenotype during their host location. Hence, we hypothesise that parasitised aphids alter HIPVs and that true aphid hyperparasitoids use altered HIPVs as a cue to locate their host.

Here, we investigated whether a true aphid hyperparasitoid can locate its parasitised aphid host through HIPVs. Hence, we used a wild *Brassica* system in which HIPVs are known to play an important role in mediating trophic interactions (Poelman *et al*., 2009). We compared the two most abundant aphid species in brassicaceous food webs and their most prevalent trophic interactions as observed through extensive field collections in a previous field study on the *Brassica oleracea* system (Bukovinszky *et al*., 2008). We tested the preference of the dominant true hyperparasitoid in this system *Alloxysta fuscicornis* (Hymenoptera: Figitidae) towards (1) uninfested control plants, (2) aphid‐infested plants and (3) parasitised aphid‐infested plants of both aphid species and measured the volatiles in the headspace of these (infested) plants. Furthermore, we measured the induced plant transcriptomic response and studied aphid feeding behaviour after parasitism to unravel how parasitism affects aphid–plant interactions that provide hyperparasitoids with cues to locate parasitised aphids.

Demonstrating that aphid hyperparasitoids can exploit parasitoid‐modified plant cues would represent a novel and striking case of convergent evolution in host‐location strategies across feeding guilds (Cusumano *et al*., 2025). Beyond its fundamental importance, understanding these mechanisms is also crucial for applied ecology. Push‐pull strategies, harnessing the chemical ecology of hyperparasitoids, have been proposed to control them (Cusumano *et al*., 2020). Although pivotal for developing such strategies, the mechanisms underlying long‐range host location by aphid hyperparasitoids are currently poorly understood. Our study addresses this gap by combining behavioural, chemical and transcriptomic approaches to link hyperparasitoid behaviour with underlying mechanisms and highlights the role of plant transcriptomic responses in shaping multitrophic interactions.

## Materials and Methods

### Plant material

A wild population of *B. oleracea* L. ‘Kimmeridge’ plants was used throughout all experiments. The original seeds of this wild population were collected from several plants near Swanage, Dorset in the United Kingdom (50.36′N, 2.07′W) (Gols *et al*., 2008). This brassicaceous plant is a wild relative to modern commercial cabbage varieties such as kale, cauliflower and broccoli. The studied plant population was propagated in the Netherlands in an open field with multiple wild *B. oleracea* genotypes by open pollination, which may have included genetic contributions from other local wild relatives as well as cultivated *B. oleracea* crops, although these were not observed in the direct neighbourhood of the field. Kimmeridge is known to have a strong induced response to herbivory, and its plant volatiles are an important source of information for insects of higher trophic levels in the food web (Gols *et al*., 2011; Zhu *et al*., 2015, 2018; Cusumano *et al*., 2018). Plants were grown in 2‐l pots containing potting soil (Lentse potgrond, Lent, the Netherlands) under glasshouse conditions (22 ± 2°C, 50–70% RH, with a 16 h : 8 h, light : dark cycle). Lighting from high‐pressure mercury lamps was used in the glasshouse to supplement periods of low natural light (below 500 μmol photons m^−2^). Plants were watered every other day, and when they were 5 wk, they were used for experiments.

### Insects and rearing

**Fig. 1:**
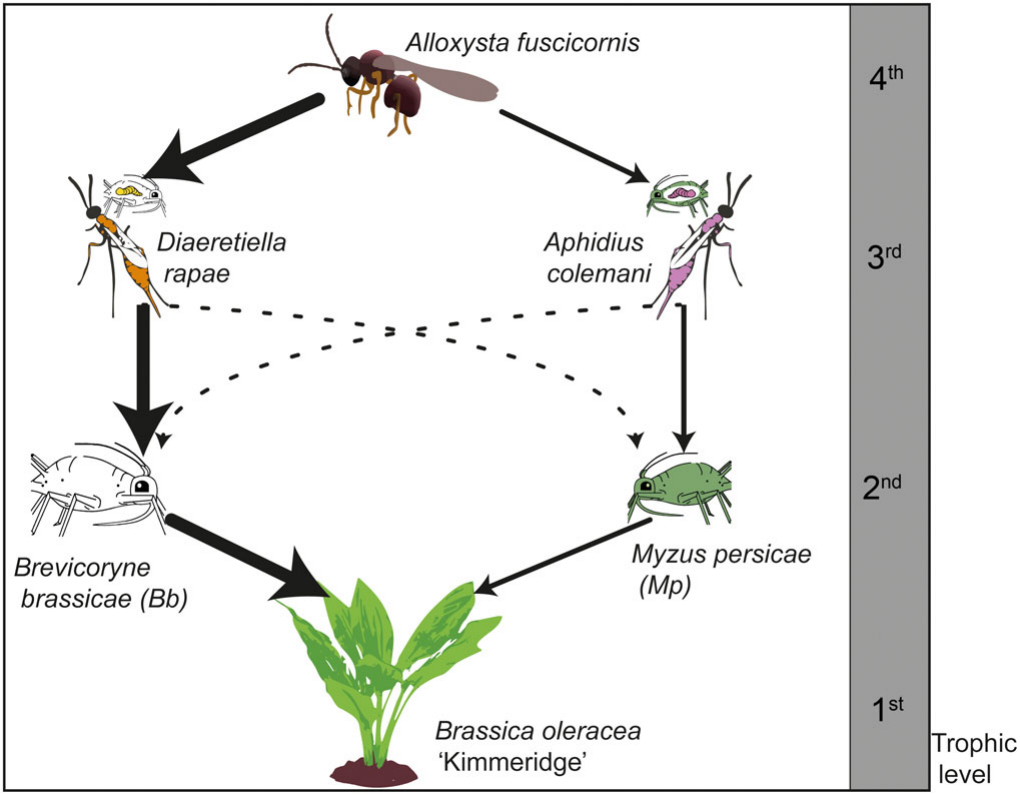
Studied food web with four trophic levels. We used a naturally occurring food web in brassicaceous systems, including the plant *Brassica oleracea* ‘Kimmeridge’ as a wild relative to modern commercial cabbage varieties and the most common interaction structures for phloem‐feeding herbivores, as observed in previous field studies on this system (Bukovinszky *et al*., 2008). Arrows indicate the direct species interactions, and arrow thickness indicates how often the interaction occurs in a natural environment. Dotted arrows indicate incidentally occurring interactions.

We studied a *Brassica* food web in which both bottom‐up and top‐down effects are known to affect food‐web structure and trophic interactions (Bukovinszky *et al*., 2008; Kos *et al*., 2011). Here, aphids are commonly found to be attacked by parasitoid wasps, which are, in turn, parasitised by hyperparasitoids (Sullivan & Völkl, 1999; Poelman *et al*., 2022). In the field, Kimmeridge plants are colonised by a wide variety of herbivores with different feeding styles (Stam *et al*., 2018). We used the two dominant aphid species, *Brevicoryne brassicae* L. (Bb) and *Myzus persicae* Sulz. (Mp) (both Hemiptera: Aphididae), in combination with their most prevalent parasitoid interactors. These are the braconid wasps *Diaeretiella rapae* McIntosh (Hymenoptera: Aphidiinae) for *B. brassicae* and *Aphidius colemani* Viereck (Hymenoptera: Aphidiinae) for *M. persicae* (Bukovinszky *et al*., 2008) (Fig. 1). As our focal study organism, we used *Alloxysta fuscicornis* Hartig (Hymenoptera: Figitidae), the most abundant true hyperparasitoid in this system (Bukovinszky *et al*., 2008). Due to its time limitation and limited host range (Brodeur, 2000; Van Veen *et al*., 2003), it is hypothesised that this hyperparasitoid uses long‐range, host‐associated cues such as HIPVs to locate its parasitised aphid host (Poelman *et al*., 2022).

Both aphid species were obtained from stock cultures maintained at the Laboratory of Entomology, Wageningen University and Research, and originally obtained from agricultural fields in the near vicinity of Wageningen, the Netherlands. Both aphid species were reared on Brussels sprouts (*Brassica oleracea* L. var. *gemmifera* cv Cyrus) plants in a glasshouse compartment at 22 ± 2°C, 50–70% RH, with a 16 h : 8 h, light : dark cycle. The parasitoid wasp *D. rapae* was originally obtained from agricultural fields in the near vicinity of Wageningen University and Research, identified through morphology (Powell, 1982) and reared on *B. brassicae* aphids on Brussels sprout plants. *A. colemani* was originally obtained as mummies (Aphipar, Koppert B.V, the Netherlands) and maintained on *M. persicae* aphids on Brussels sprouts plants. The true hyperparasitoid *A. fuscicornis* was originally obtained in 2019 from the strip cropping fields near Wageningen University and Research, the Netherlands, identified to the genus level morphologically (Ferrer‐Suay *et al*., 2013), and finally to the species level by sequencing the internal transcribed spacer (ITS2) region (Van Veen *et al*., 2003). *A. fuscicornis* was reared on *B. brassicae* aphids on Brussels sprouts plants, which were priorly exposed to *D. rapae* parasitism for 2 d. Parasitoids were kept in a climate cell at 22 ± 1°C, 50–70% RH and 16 h : 8 h, light : dark cycle. Adult *A. fuscicornis* were kept in a rearing cage at room temperature, under natural light conditions (22°C ± 2°C, 35–45% RH). All (hyper)parasitoid wasps were weekly supplied with a 1 : 10 honey: water solution and water.

### Aphid parasitism treatment

**Fig. 2:**
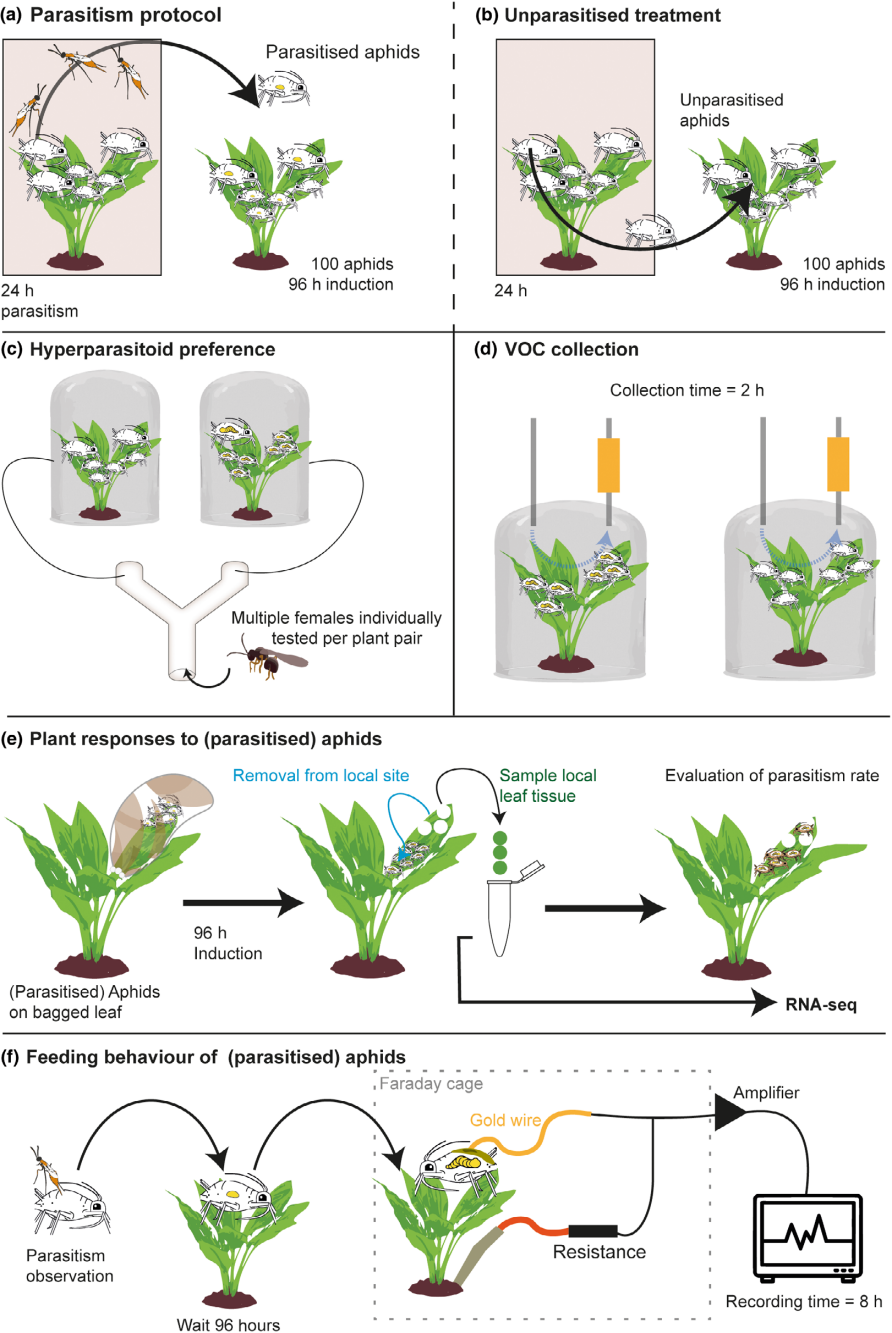
Workflow of this study. (a) We induced *Brassica oleracea* ‘Kimmeridge’ plants for 96 h with parasitised aphids, (b) unparasitised aphids or uninduced control plants. (c) These plants were used to study hyperparasitoid preference in a Y‐tube olfactometer in which multiple female hyperparasitoids were individually tested per plant pair on a day (*n* = 9–10 plant pairs per combination) or (d) to collect the volatiles from their headspace, *n* = 11 biological replicates per treatment, each replicate consisting of volatiles collected from a single plant. (e) Furthermore, plants were induced with (parasitised) aphids on a bagged leaf for 96 h. Local leaf‐feeding sites were sampled, RNA isolated and sequenced, *n* = 5 biological replicates per treatment, each replicate consisting of pooled leaf tissue from eight different plants. (f) The feeding behaviour of parasitised and unparasitised aphids was studied with the electrical penetration graph method (*n* = 30 aphids tested per treatment).

**Fig. 3:**
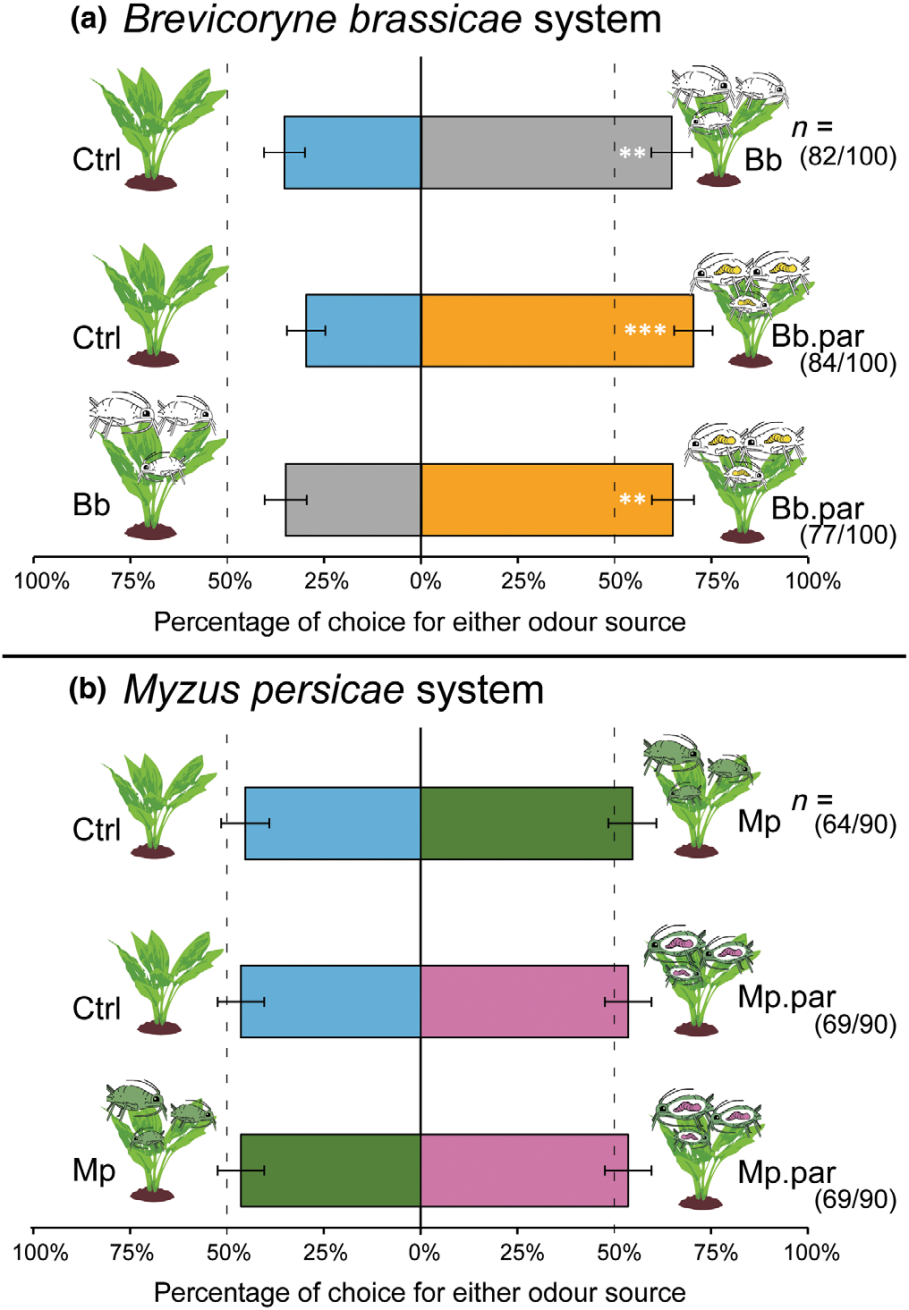
Preference of the aphid hyperparasitoid *Alloxysta fuscicornis* to odours of plants induced with (parasitised) aphids. (a) Preference for unparasitised and parasitised aphid‐induced plants for the naturally most prevalent specialist *Brevicoryne brassicae* (Bb and Bb.par) and (b) the less prevalent generalist *Myzus persicae* (Mp and Mp.par). Both aphid species were parasitised by their specialist parasitoid. Bb.par, *B. brassicae* parasitised by parasitoid *Diaeretiella rapae*. Mp.par, *M. persicae* parasitised by its parasitoid *Aphidius colemani*. N indicates the number of hyperparasitoids making a choice/total individually tested wasps in the olfactometer per tested treatment combination. In total, 10–11 plant pairs were tested per treatment combination. Each plant pair was tested with multiple naïve hyperparasitoid females (depending on hyperparasitoid availability; each female used only once). Asterisks indicate: **, *P* < 0.01 and ***, *P* < 0.001 for a binomial generalised linear model (GLM). Error bars indicate the SE of the mean.

Previous research showed that caterpillar parasitism affects induced plant responses (Poelman *et al*., 2011b; Tan *et al*., 2018), plant‐mediated interactions (Cusumano *et al*., 2018) and leads to the attraction of hyperparasitoids (Zhu *et al*., 2018). To study the effect of parasitised aphids on these interactions, we first (1) exposed Brussels sprouts plants to one aphid species for 24 h to their corresponding parasitoid by placing them together in a cage with a 10 : 1 aphid–parasitoid ratio (Fig. 2a) to obtain parasitised aphids (Bb.par or Mp.par). Or, (2) simultaneously placed plants with aphids in a cage without parasitoids to obtain unparasitised aphids (Fig. 2b) (Mp or Bb). After 24 h, these plants were removed from the cages, and their aphids were transferred to induce Kimmeridge plants. The induction lasted for 96 h after which the plants were subjected to an experimental procedure as (1) hyperparasitoid preference in the Y‐tube olfactometer (Fig. 2c), (2) collection of volatile organic compounds (VOCs) (Fig. 2d) or (3) sampling of leaf material to study the transcriptomic response (Fig. 2e). As a control treatment, we included plants that were handled in the same way but were not induced with aphids (Ctrl).

### Y‐tube olfactometer bioassays

We studied in a Y‐tube olfactometer set‐up whether the volatiles of (parasitised) aphid‐induced plants are used by *A. fuscicornis* to locate aphid‐infested plants over uninfested plants. In addition, we studied whether *A. fuscicornis* can differentiate between the volatiles of plants infested with their parasitised aphid host vs unparasitised aphid‐infested plants of the same aphid species (Bb.par vs Bb and Mp.par vs Mp). Each plant pair was tested with multiple naïve female hyperparasitoids (depending on hyperparasitoid availability, usually 8–10), with each female used only once. Per tested combination, we used 9–10 plant pairs. This procedure is described in detail in Supporting Information Methods S1.

### Collection and analysis of volatile organic compounds

Headspace samples were collected from 11 plants per treatment, resulting in a total of 55 samples. Headspace samples were collected in 30‐l glass jars sealed with a rubber‐lined glass lid containing an inlet for clean air. Volatiles emitted by the plants were trapped by drawing air out of the glass jar at a suction rate of 200 ml min^−1^ through a stainless steel tube filled with 200 mg Tenax TA (20/35 mesh; CAMSCO, Houston, TX, USA) for 2 h. This is described in more detail in Methods S2.

### Transcriptomics of (parasitised) aphid‐induced leaf samples

#### Insect treatments and induction of plants

To prepare parasitised aphids for the induction treatments, Brussels sprouts plants with ±1000 aphids were placed in a mesh cage (60 × 40 × 40 cm, Ant's Kingdom, the Netherlands) and exposed to ±100 parasitoid adults for 48 h. Due to the commercial unavailability of *D. rapae* and the requirement for a large number of parasitoids, *B. brassicae* was parasitised by a freshly obtained, wild parasitoid population, which was visually assessed to mainly consist of *D. rapae*. This population was obtained by exposing *B. brassicae*‐infested Kimmeridge (±1000 aphids) plants to parasitism in the field for 3 d and collecting the emerged parasitoids. According to previous studies, the *B. brassicae*‐associated parasitoid population mainly consists of *D. rapae* (Bukovinszky *et al*., 2008; Derocles *et al*., 2023). *M. persicae* was parasitised by newly emerged *A. colemani* (Koppert, the Netherlands). Parasitoids were also supplied with honey water (1 : 10) and water. Unparasitised aphids used during the experiment were maintained in the bordering glasshouse compartment under the same conditions.

To ensure a homogenous induction, Petri dishes (diameter = 35 mm) with 25 apterous adult aphids were prepared a few hours before induction and sealed with parafilm. We enclosed the youngest fully expanded leaf in a mesh drawstring bag with an open end. Induction of plants was performed by placing an open Petri dish upside down on the youngest fully expanded leaf, allowing the aphids to move on the plant. Control plants received Petri dishes without aphids. Directly after placing the Petri dish, the open end of the mesh bag was closed with three small wooden clothespins to restrict the aphids from moving to other leaves or plants.

#### Sampling local leaves

After 96 h of induction, each plant was sampled individually by taking three leaf discs (ø = 2 cm) from a local feeding site of aphids with a sterilised puncher. Leaf samples were placed in a 2‐ml Eppendorf tube, immediately flash‐frozen in liquid nitrogen and stored at −80°C until the parasitism status of the aphids was confirmed. Before sampling, wooden clothespins were removed to open the bag, leaves were kept attached, and aphids were moved aside gently with a brush. For parasitised aphids, the bags were closed again after sampling. To ensure parasitism, parasitised aphid‐induced leaves were checked 1 wk after sampling for the presence of aphid mummies. Parasitised aphid‐induced samples were included when more than nine aphid mummies were present. On average, the included samples of Bb.par had 12.4 mummies and Mp.par had 12.9 mummies, corresponding to an overall parasitism rate of *c*. 50.4% (Fig. S1; Table S1).

#### RNA isolation and sample preparation for RNA sequencing

Frozen leaf samples were ground to a fine powder with a sterile pestle. Per treatment, five biological replicates were created by pooling an equal amount of ground leaf material from eight different plants. Plants represented in a biological replicate were randomly distributed in the glasshouse. To minimise variation in parasitised treatments caused by a difference in parasitism success, we balanced the average number of mummies in each biological replicate (Table S1). More detail on the extraction protocols is described in Methods S3.

#### Quality control and read processing

Described in Methods S3.

#### Differential gene expression analysis

Obtained read counts were processed in R (v.4.2.0) using the DESeq2 package (Love *et al*., 2014; Karssemeijer *et al*., 2022). In short, we removed low‐count genes (< 10 counts on average across all samples) and calculated differentially expressed genes (DEGs) through a model with a combined factor for all treatments. This model was relevelled to the uninduced control treatment as baseline, and genes were classified as DEG if they were different from the control with a false discovery rate lower than 0.01 and an absolute log_2_‐fold change > 0.5. To preserve true, large differences across conditions in the estimated logaritmic fold changes (LFCs) we used apeGLM as shrinkage estimator (Zhu *et al*., 2019). The number of unique and overlapping DEGs between different treatments was visualised in an upSet plot (Li, 2023).

#### Multivariate analysis

Regularised logarithm transformed counts (rlog counts) (Love *et al*., 2014) were used as input for principal component analysis (PCA), using the PCAexplorer package in R with standard settings (Marini & Binder, 2019) and visualised with ggplot2 (Wickham, 2016). Genes in the top and bottom loadings of PC1 were extracted using the ‘ntop’ command of PCAexplorer. The closest *Arabidopsis* homologue of these genes was characterised using PLAZA 4.5 (Van Bel *et al*., 2018). Their function was assigned using descriptions in the TAIR database (www.arabidopsis.org). If gene function was unknown from this database, we used the UNIPROT database to annotate the gene function based on protein structure.

To test whether gene expression differed between treatments, inducers and parasitism status, we used the ‘adonis’ function in Vegan to perform a PERMANOVA (Oksanen *et al*., 2015). For this purpose, we calculated a Bray–Curtis distance matrix based on rLog values of each sample for the top 500 unique genes with the highest or lowest loading scores in PC1 and PC2. Significance was tested using 1000 permutations. We used two models, of which the first included treatment as the sole factor. This was followed by pairwise PERMANOVAs to compare the expression of individual treatments using the pairwise.adonis2 function of the pairwise.Adonis package (Martinez Arbizu, 2020). For the second model, the control was omitted from the dataset to include the factors ‘inducer identity’, ‘parasitism rate’ and their interaction factor in the model.

#### K‐means clustering

Log‐transformed counts of all DEGs were mean‐centred and scaled per gene to obtain *Z*‐scores. K‐means clustering was performed using the R function kmeans () (R Core Team, 2024), with 30 iterations and seven clusters. Clusters were subjected to Gene Ontology (GO) enrichment analysis using PLAZA 4.5, with the total set of expressed genes (29 999 genes) as background (Van Bel *et al*., 2018). Line plots were generated by computing the average log_2_‐transformed counts (log_2_(count +1)) for each gene per treatment group, followed by *z*‐score normalisation across treatments. These normalised expression profiles were grouped by K‐means cluster and plotted per cluster.

#### Pathway analysis

Expression of genes involved in defence‐related pathways (SA, JA, ET and abscisic acid (ABA)) was characterised (Karssemeijer *et al*., 2022). Several pathogenesis‐related (PR) genes and WRKY transcription factor family (WRKY) genes were selected based on earlier studies (Singh *et al*., 2018). Furthermore, we characterised genes involved in terpene pathways and synthesis as known in the literature (Tholl & Lee, 2011; Hofberger *et al*., 2015; Tholl, 2015) and identified orthologous genes in *B. oleracea* using the integrative orthology viewer of PLAZA 4.5 (Van Bel *et al*., 2018; Karssemeijer *et al*., 2022).

### Recording (parasitised) aphid feeding behaviour with the electrical penetration graph (EPG)

To investigate the feeding behaviour of (un)parasitised *B*. *brassicae* and *M*. *persicae* aphids, the electrical penetration graph (EPG) technique was employed (Tjallingii, 1988). In short, a golden wire (18 μm diameter and 1.5 ± 0.5 cm in length) was gently attached to the dorsum of apterous aphid adults using water‐based silver glue, aphids were tethered to the 2^nd^ true leaf of a Kimmeridge plant, and an electrode was placed in the potting soil of the plant. This was placed in a Faraday cage to reduce electrical noise (Fig. 2f). The voltage fluctuations produced during aphid feeding were amplified using Direct Current Giga‐8 systems (http://www.epgsystems.eu), recorded for 8 h with the EPG Stylet+ software (www.epgsystems.eu), the corresponding feeding behaviour was annotated according to their waveform patterns, and data were analysed using R v.4.0.4 (Kloth *et al*., 2021), with the adaptations that nonexisting waveforms were assigned as zero values for total duration and as missing values for mean values. Behaviours that were interrupted by the end of a recording were included in the analyses. Each replicate was obtained by one aphid feeding on one plant.

#### Preparing aphid treatments for EPG

To obtain parasitised aphids (Bb.par and Mp.par) for the EPG experiment, 5–10 apterous aphids, a Kimmeridge leaf, and 2–5 mated parasitoid females were released inside a Petri dish. In line with previous experiments, we used *D. rapae* for *B. brassicae* aphids and *A. colemani* for *M. persicae* aphids (Fig. 1). After observing a successful event of parasitism, the parasitised aphid was transferred to a Kimmeridge plant and kept there for 96 h before the recordings (Fig. 2f). For the control group, aphid adults were picked from the same batch as parasitised aphids but directly placed on Kimmeridge plants until recording. After the EPG recordings finished, the parasitised aphids were untethered and kept on their respective plant for an additional day. We only included parasitised aphids in the analysis when we successfully confirmed their parasitism status through dissection. In case no parasitoid larva was present in the parasitised aphid, the recording data were omitted and not used in any analysis.

## Results

### Aphid hyperparasitoid successfully locates plants with parasitised *B. brassicae* aphids

A total of 570 female *A. fuscicornis* hyperparasitoids were tested in two‐choice Y‐tube olfactometer tests, with an average response rate of 77.9% (ranging between 71 and 84% for different combinations; between 90 and 100 females tested per treatment combination). The hyperparasitoids were offered the odours of plants with 100 (parasitised) aphid adults of the species *B. brassicae*, *M. persicae* or uninduced control *B. oleracea* ‘Kimmeridge’ plants. Before testing, these plants were induced for 96 h with their respective treatment (Fig. 2a–c). The hyperparasitoids preferred volatiles of plants with parasitised *B. brassicae* aphids over volatiles of plants with unparasitised aphids (Wald *Z* = 2.580; *P* = 0.010) or uninduced control plants (Wald *Z* = 3.598; *P* < 0.001). In addition, odours of plants induced with unparasitised *B. brassicae* aphids were preferred over uninduced control plants (Wald *Z* = 2.611; *P* = 0.009) (Fig. 3a). However, this phenomenon was found to be aphid‐specific and did not hold true for plants induced with the aphid species *M. persicae* that are less prevalent on Brassicaceae plants. The hyperparasitoid did not exhibit a preference for plants induced with parasitised *M. persicae* over unparasitised *M. persicae* aphids (Wald *Z* = 0.601; *P* = 0.548) or undamaged control plants (Wald *Z* = 0.601; *P* = 0.548). In addition, no preference was found for plants with unparasitised aphids against undamaged control plants (Wald *Z* = 0.749; *P* = 0.454) (Fig. 3b).

The preference of the aphid hyperparasitoid for plants induced with (parasitised) *B. brassicae* aphids indicates that feeding by parasitised *B. brassicae* triggers the interaction chain, which ultimately allows the hyperparasitoid to locate its host. This is often mediated by qualitative and quantitative alterations in the emission of plant volatiles (Turlings & Erb, 2018). Hence, we collected the headspace of plants with the treatments as mentioned previously (Fig. 2d), although parasitism rates in this experiment were unfortunately much lower (parasitism rate < 0.25) than those in other experiments (average parasitism rate = 0.46) (Fig. S1; Tukey's HSD: *P* < 0.001). A total of 50 VOCs were identified, which are categorised in nine different chemical classes (Fig. S2b). Multivariate analyses on the obtained VOC peak heights did not yield models with explanatory value (PLS‐DA: R2X = 0.421, R2Y = 0.0959, Q2 = −0.119; Table S2). In addition, amounts (in terms of peak heights) of none of the compounds were affected by the different treatments (Fig. S2a).

As an alternative approach to analyse the volatile data, that exhibited very large variation, we used a DESeq2 approach (Love *et al*., 2014; Zhu *et al*., 2019). This approach should be regarded as a complementary way of diving deeper into a dataset that otherwise did not reveal clear patterns. Differentially emitted VOCs were identified between several treatments and control. This analysis indicated that (*E*)‐4,8‐dimethyl‐1,3,7‐nonatriene ((*E*)‐DMNT) is slightly, but significantly upregulated for parasitised *B. brassicae* (Bb.par), and several sesquiterpenes are reduced compared to uninfested control plants (Tables S3, S4). This was not the case when *B. brassicae* were unparasitised (Bb). We identified methyl thiocyanate as characteristic compound for plant responses to parasitised *M. persicae* (Mp.par). This suggests that there are subtle differences in the volatile blend, which are hard to detect with our quantification method and multivariate statistics. Moreover, the hyperparasitoid may use subtle differences in volatile blends that are likely associated with changes in compound ratios or compounds that are difficult to detect with our instrument, collection method and/or analytical tools.

### Aphid species and parasitism status affect plant transcriptomic responses

**Fig. 4:**
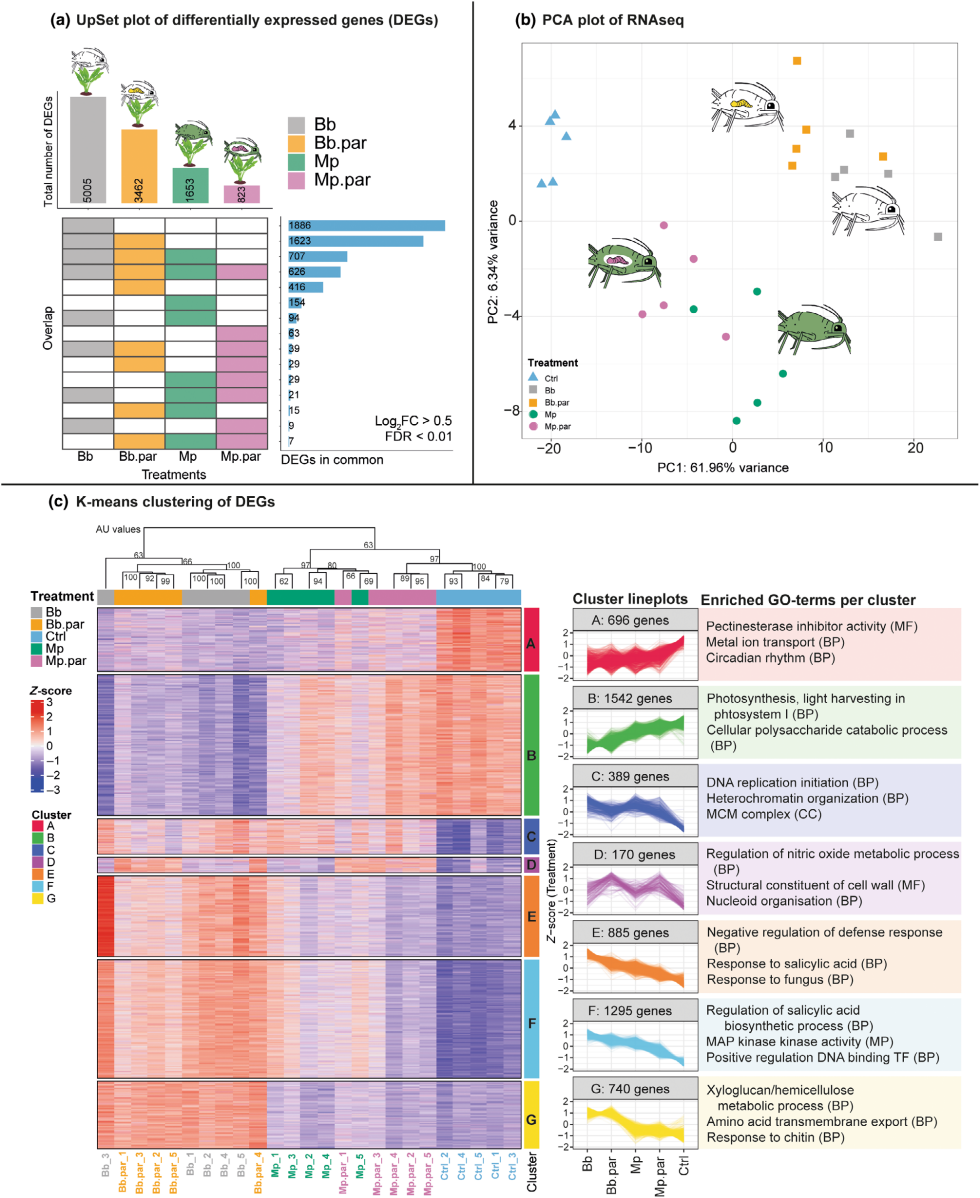
General *Brassica oleracea* ‘Kimmeridge’ plant transcriptomic response to feeding by (parasitised) aphids of the species *Brevicoryne brassicae* and *Myzus persicae*. (a) UpSet plot of differentially expressed genes (DEGs with a false discovery rate < 0.01, and log_2_ fold change (log_2_FC) > 0.5 or < −0.5). (b) Principal component analysis plot of plant responses to all treatments. (c) K‐means clustering of DEGs with line plots per cluster and enriched Gene Ontology terms. For hierarchical clustering, *P*‐values were calculated with multiscale bootstrap resampling (10 000 bootstraps) based on approximately unbiased (AU) probability. Here, clusters with AU‐probability larger than 95 are strongly supported by data. AU values are indicated on the branches. Both aphid species were parasitised by their specialist parasitoid. Used abbreviations: Bb.par, *B. brassicae* parasitised by parasitoid *D. rapae*; BP, GO terms from the class ‘biological product’; CC, GO terms from the class ‘cellular component’; DEG, differentially expressed gene; MF, GO terms from the class ‘molecular function’; Mp.par, *M. persicae* parasitised by its parasitoid *A. colemani*. *n* = 5 biological replicates per treatment, each replicate consisting of pooled leaf tissue from eight plants.

Because the preference of higher trophic levels is often associated with the induced plant response after herbivore feeding, we characterised how the plant transcriptome was altered by aphid species and their parasitism status. We induced confined leaves of plants with 25 (parasitised) aphids of either species or kept uninduced control plants for 96 h, followed by the removal of the aphids and sampling of the plant material at the local feeding site (Fig. 2e). In total, we found 5718 DEGs between uninduced control plants and any of the treatments (Fig. 4a). Most of these genes were modulated after induction with *B. brassicae*, which was also the treatment with the most unique DEGs. Furthermore, a core of 626 genes was found to be altered upon any aphid induction.

A lower number of DEGs were identified in plants induced by parasitised aphids, compared to their unparasitised counterparts for both aphid species. Induction with *M. persicae* (1653 DEGs in total) led to a three times lower number of DEGs compared to *B. brassicae* (5005 DEGs in total), with only a few unique DEGs. Multivariate analysis revealed clear patterns in the transcriptomic profiles upon induction with (parasitised) aphids. In addition to treatment (*F*_4,20_ = 27.202, *P* < 0.001), inducer identity (PERMANOVA: *F*_1,16_ = 34.0717, *P* < 0.001), the presence/absence (binary) of parasitised aphids (*F*_1,16_ = 9.3893, *P* = 0.006) and the observed parasitism rate (*F*_1,16_ = 9.3774, *P* = 0.002) were shown to be significant factors in a reduced model (removing control treatment) (Table S2b). Furthermore, a pairwise PERMANOVA indicates that all treatments were significantly different from one another (Table S5). Parasitism status and inducer identity were not found to have an interaction (*F*_1,16_ = 33.3761; *P* = 0.886); parasitised treatments cluster closely with their unparasitised counterparts in the PCA. Here, the first PC, which explains 61.96% of the variation, separates parasitised treatments from unparasitised treatments and aphid‐induced plants from the control. This separation was clearer for *M. persicae*, while for *B. brassicae*, parasitised and unparasitised treatments clustered more closely together in the upper right quadrant. The second PC, explaining 6.34% of the variation, mainly separates the two different aphid species (Fig. 4b). We functionally characterised the genes that contributed most to the separation on PC1 and PC2. These mainly include genes involved in induced plant defence responses (*PR‐*genes, *WRKY38*, *CHI‐*genes and *VSP2*) and cell‐wall‐related genes (*PME‐*genes, Extensins or extensin‐like proteins, *BXL*) (Tables S6, S7).

### Salicylic acid‐ and ethylene‐related genes are the main players in modulating the response against (parasitised) aphids

Parasitism often changes herbivore physiology and consequently induces plant responses. Direct defences to herbivory are mediated by the biosynthesis and regulation of various defence‐related phytohormones. Especially SA, known to be an important mediator of aphid‐induced plant responses (Pieterse *et al*., 2012), is abundant in the enriched GO terms of the clustering analysis (Fig. 4c, cluster E & F) and bottom/top loadings in PC1 and 2 (Tables S6, S7).

**Fig. 5:**
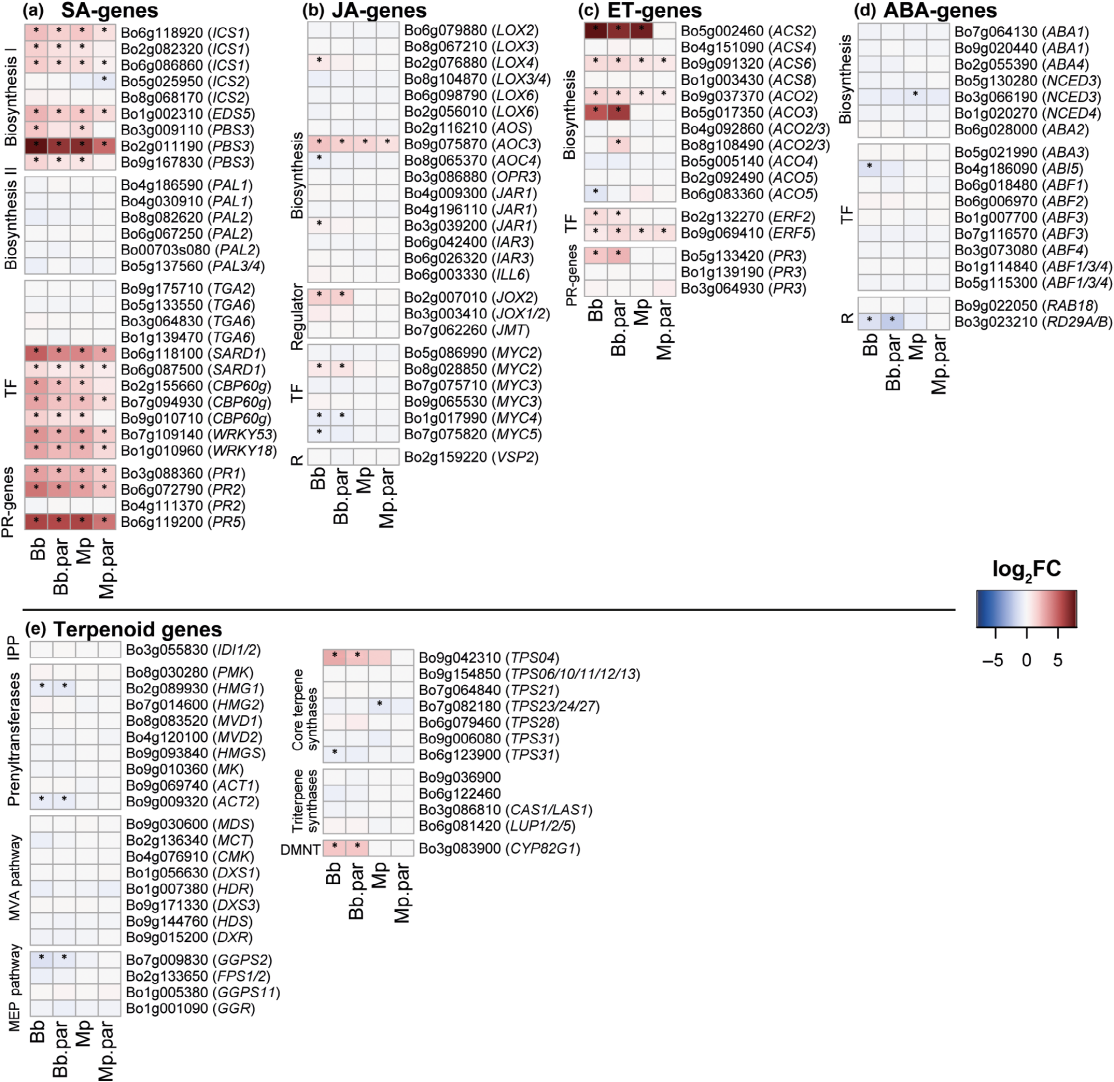
*Brassica oleracea* ‘Kimmeridge’ plant responses for genes in defence‐related pathways, 96 h after induction with respective (parasitised) aphid induction treatment of the species *Brevicoryne brassicae* and *Myzus persicae*. (a) Salicylic acid (SA) pathway. (b) Jasmonic acid (JA) pathway. (c) Ethylene pathway (ET). (d) Abscisic acid pathway (ABA). (e) Genes related to terpenoid biosynthesis. Asterisks indicate differentially expressed genes compared to uninduced control plants with false discovery rate < 0.01 and absolute Log_2_FC > 0.5, calculated with DESeq2. Used abbreviations: DMNT, 4,8‐dimethyl‐1,3,7‐nonatriene; IPP, isopentenyl diphosphate; MEP, methylerythritol phosphate; MVA, mevalonate; PR, pathogenesis‐related; TF, transcription factor. Both aphid species were parasitised by their specialist parasitoid. Bb.par, *B. brassicae* parasitised by parasitoid *Diaeretiella rapae*. Mp.par, *M. persicae* parasitised by its parasitoid *Aphidius colemani*. *n* = 5 biological replicates per treatment, each replicate consisting of pooled leaf tissue from eight different plants.

In general, most genes in the SA‐pathway are stronger upregulated in response to unparasitised aphids compared to parasitised aphids of the same species (Fig. 5a). Most genes involved in the biosynthesis of SA through the isochorismate (ICS) pathway were significantly upregulated for all treatments, except for *ICS2*, which was downregulated as a response to parasitised *M. persicae* (Mp.par). Positive defence regulators (*WRKY53* and *WRKY18*) and pathogenesis‐related (*PR*) resistance genes were upregulated in all treatments but were generally stronger expressed for unparasitised aphids than for parasitised aphids. A gene identified as *PR2* homologue was much stronger (Log_2_FC = 7.99) upregulated in response to infestation by parasitised *B. brassicae* compared to unparasitised *B. brassicae* (Fig. S3; Tables S8, S9).

Along with the SA pathway, various ET biosynthetic genes and transcription factors were upregulated in aphid‐infested plants (Fig. 5c). This was stronger for *B. brassicae‐induced* plants than those induced with *M. persicae*. *ACO2/3* upregulation was only found in plant responses to parasitised *B. brassicae* (Bb.par) and downregulation of one gene involved in the same step of ET production was found for plant responses to unparasitised *B. brassicae* (Bb). Furthermore, pathogenesis‐related gene *PR3* is only differentially expressed upon induction with *B. brassicae* aphids. Contrasts between parasitised and unparasitised aphids of the same species indicate that only one gene in this pathway (*ACO2/3*) is differentially expressed between (un)parasitised *B. brassicae* aphids (Fig. S3).

In contrast to SA and ET, both JA and ABA pathways appear only slightly altered upon induction with (parasitised) aphids (Fig. 5b,d). Neither pathway exhibited a clear response to treatment with *M. persicae*. When plants are exposed to *B. brassicae* treatments, gene expression appears to be mainly targeted at suppression of the JA and ABA pathways or even at halting a response. For example, jasmonate‐induced oxgenase‐2 (*JOX2*) is upregulated in plants infested with *B. brassicae* (Bb and Bb.par). This gene is known to divert JA from conversion to its bio‐active form JA‐Ile (Smirnova *et al*., 2017).

### Only homoterpene‐related genes are upregulated in the terpenoid pathway

Terpene‐derived compounds are known to impact trophic interactions after herbivory (Kappers *et al*., 2005). These are slowly produced and diffused and can act as highly specific cues (Escobar‐Bravo *et al*., 2023). Few genes in terpenoid pathways were found to be differentially expressed after (parasitised) aphid induction, mostly by (parasitised) *B. brassicae* (Fig. 5e). Two genes involved in homoterpene synthesis, terpene synthase 4 (*TPS4*) and *CYP82G1*, (Attaran *et al*., 2008; Tholl *et al*., 2011) were upregulated for *B. brassicae*‐infested plants but not for *M. persicae*‐infested plants. In addition, several genes further upstream were found to be downregulated after induction with (parasitised) *B. brassicae*. This includes the gene *GGPS2*, which synthesises geranylgeranyl pyrophosphate, which is catabolised by TPS4 (Attaran *et al*., 2008). For *M. persicae*‐infested plants, only one gene (*TPS23/24/27*), likely involved in monoterpene biosynthesis (Tholl & Lee, 2011), was affected and found to be downregulated. No effect of parasitism was found on terpenoid‐biosynthesis‐related genes (Fig. S3).

### Aphid feeding behaviour is only slightly affected by parasitism

**Fig. 6:**
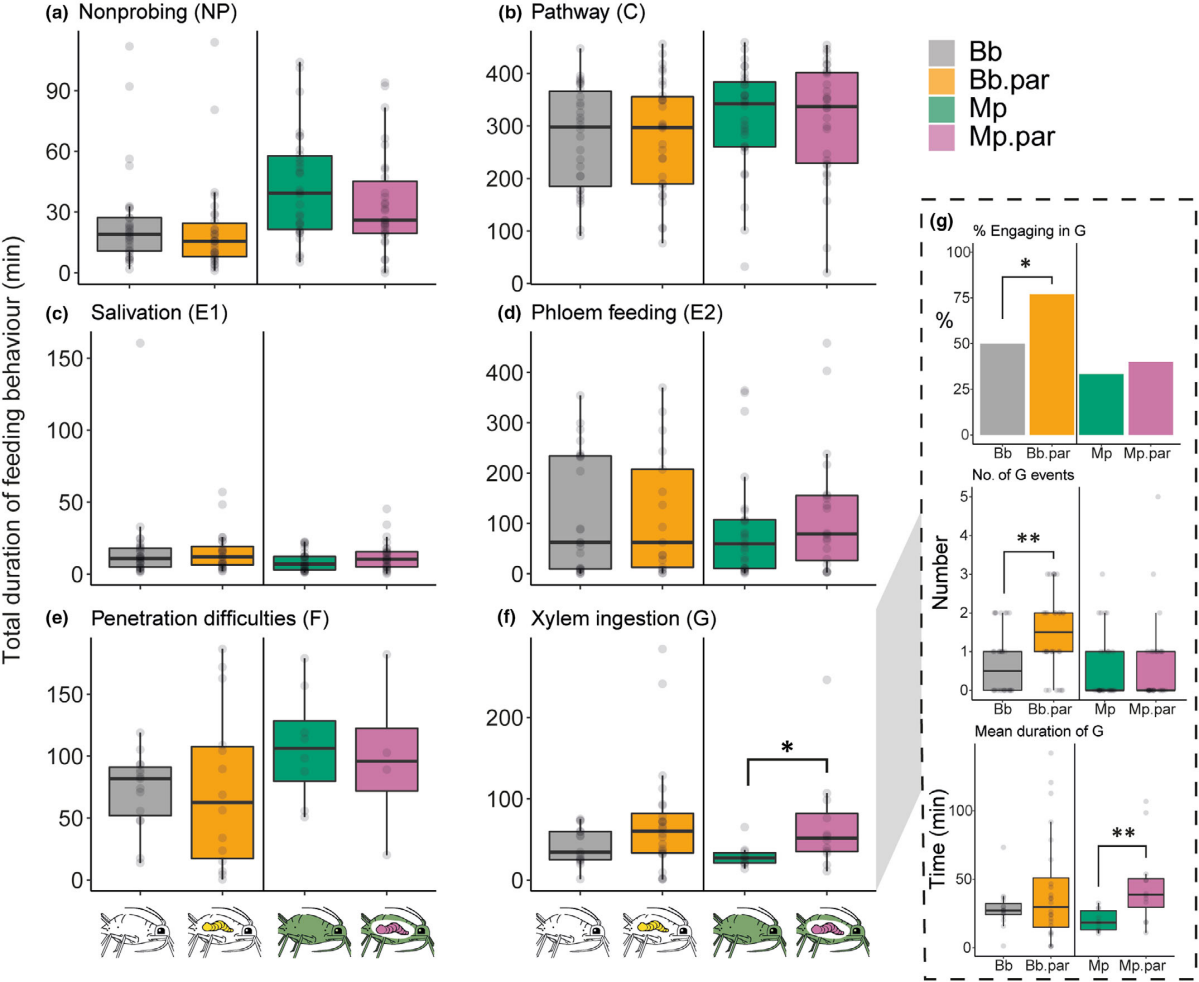
Total duration of the main parameters in aphid feeding behaviour (a) Nonprobing, (b) pathway, (c) salivation, (d) phloem feeding, (e) penetration difficulties, (f) xylem drinking, (g) subparameters of xylem drinking including (1) % of aphids engaging in G, (2) number of G events, (3) mean duration of G. *, *P* < 0.05; **, *P* < 0.01 for Mann–Witney *U*‐test. Each boxplot shows the median (horizontal line), the interquartile range (IQR; box limits), and whiskers extending to 1.5× IQR. For each treatment, *n* = 30 aphids were individually tested, the number of aphids performing each behaviour is given in Supporting Information Table S10. Both aphid species were parasitised by their specialist parasitoid. Bb.par, *Brevicoryne brassicae* parasitised by parasitoid *Diaeretiella rapae*. Mp.par, *Myzus persicae* parasitised by its parasitoid *Aphidius colemani*. The white aphids in the figure represent *B. brassicae* and the green aphids *M. persicae*.

Plants induced with parasitised aphids show similar, but attenuated, responses compared to induced responses to unparasitised aphids of the same species. We studied the aphid feeding behaviour to pinpoint whether this difference can be linked to changes in the aphid feeding behaviour after parasitism. Our results indicate that there are no large differences in the total duration of five out of six main feeding parameters between parasitised and unparasitised aphids of the same species (Fig. 6a–e; Table S10). However, parasitised *M. persicae* aphids ingested xylem sap significantly longer than unparasitised aphids of the same species (U = 28.0, *P* = 0.036; Fig. 6f), and similarly, *B. brassicae* interacted more often with the xylem when parasitised. For the latter species, parasitised aphids showed an increased number of xylem drinking events (*U* = 265.5, *P* = 0.004) and an increased percentage of aphids engaging in xylem drinking when parasitised (χ^2^ = 4.69, *P* = 0.030) (Fig. 6g).

## Discussion

The aphid hyperparasitoid *A. fuscicornis* can locate its hidden parasitoid host by using volatile cues from plants colonised by parasitised aphids in a brassicaceous system. Specifically, when induced by the most abundant aphid–parasitoid interaction in its food web (*B. brassicae* parasitised by *D. rapae*), *A. fuscicornis* shows a marked preference for plants induced with (parasitised) aphids. However, similar preferences were not observed in the case of a second, less abundant (parasitised) aphid species in the food web (*M. persicae–A. colemani*). Our results suggest that the observed phenomenon is the consequence of a complex interplay of factors, including subtle variation in volatile blends, plant gene transcript levels and minor alterations in aphid feeding behaviour, which are influenced by both aphid species and parasitism (rate). The ability of hyperparasitoids to eavesdrop on plant volatiles induced by herbivory of parasitised herbivores has previously been reported in the case of hyperparasitoids in a caterpillar‐related food web of the same plant species (Poelman *et al*., 2012; Zhu *et al*., 2015; Cusumano *et al*., 2019). We extend this knowledge by demonstrating that aphid‐associated hyperparasitoids may detect and exploit subtle changes in plant phenotype caused by some parasitised aphids (*B. brassicae–D. rapae*). The similarity in the use of parasitised herbivore‐induced volatiles by aphid and caterpillar‐associated hyperparasitoids is remarkable, because aphid‐induced plant responses differ widely from caterpillar‐induced plant responses (Pieterse *et al*., 2012).

Our study revealed that plant responses to aphid herbivory, as indicated by changes in gene expression, are influenced by both aphid species and parasitism. This is in line with previous studies showing that plants can respond differentially to herbivory by different insect species of the same feeding guild (Fernández De Bobadilla *et al*., 2021), but respond more strongly to more prevalent herbivores in their natural context (Mertens *et al*., 2021). We observed a higher number of DEGs as well as higher transcript abundance in response to herbivory by the most abundant aphid species compared to a less abundant species. Our findings are also consistent with reports that parasitism can alter plant responses to herbivory (Poelman *et al*., 2011b; Tan *et al*., 2018; Vaello *et al*., 2018). In the case of induction with parasitised aphids, the number of DEGs and the intensity of their expression were reduced. However, no strong overriding effect of parasitism was observed in volatile profiles as was previously found for chewing herbivores (Zhu *et al*., 2015).

Terpenoid VOC are well‐known to be involved in indirect plant defences (Kappers *et al*., 2005; Tholl *et al*., 2011; Escobar‐Bravo *et al*., 2023) and differentially affected by the herbivory of the two aphid species. We found gene *CYP82G1*, which catalyses the production of homoterpenes (*E*)‐DMNT and TMTT (*(E,E)*‐4,8,12‐Trimethyl‐1,3,7,11‐Tridecatetraene), to be expressed upon induction with *B. brassicae*, but not *M. persicae*. This signal in the transcriptomic plant response points in the same direction as the measured volatile signal but is much stronger. This suggests that the specificity of (*E*)‐DMNT induction may contribute to the observed aphid species specificity of hyperparasitoid attraction. (*E*)‐DMNT is more often shown to increase the attractiveness of volatile blends to insects (Tasin *et al*., 2007; Mumm *et al*., 2008). The hyperparasitoid preference for the most abundant aphid species in the food web has likely evolved as a result of the higher encounter rate (Bukovinszky *et al*., 2008) or a higher profitability of this host (Dicke & Grostal, 2001; Harvey *et al*., 2015). However, we should note that *A. fuscicornis* was reared on the *B. brassicae* system for several generations before testing, which might have affected its preference for *B. brassicae*.

Rearing background is known to affect the preference of parasitoids (Bodino *et al*., 2016). Yet, the rearing background used in this study accurately represents the natural context in which *B. brassicae* parasitised by *D. rapae* is by far the most dominant host for *A. fuscicornis* (Bukovinszky *et al*., 2008). Moreover, the (hyper)parasitoids used in our study were obtained not only from the same agricultural fields as the aphids (*D. rapae* and *A. fuscicornis*) but also from a commercial supplier (*A. colemani*). It is important to acknowledge that the use of trophic interactions that did not co‐evolve could have influenced our results. Non‐co‐occurring populations may not exhibit the same adaptive responses as co‐evolved populations (Mertens *et al*., 2021), particularly in complex trophic interactions like those in this study.

How the aphid hyperparasitoid can discriminate between plants infested with (parasitised) *B. brassicae* aphids is less clear than the difference between the two studied aphid species. The transcriptomic plant response, particularly the SA‐response, appears to be attenuated after induction with parasitised aphids. In line with this, clustering analysis indicated that volatile profiles of plants induced with parasitised *B. brassicae* are more similar to uninduced control plants than plants induced with unparasitised aphids of the same species. Identifying the specific volatile compounds and the sensory mechanisms (e.g. with GC‐EAD) involved in hyperparasitoid attraction will be important for understanding the mechanistic basis of this preference (Bisch‐Knaden *et al*., 2022). Although it is likely that not individual compounds, but rather the ratio of these in the total blend is more important in the case of such intricate host location (Bruce & Pickett, 2011; Aartsma *et al*., 2019). It is important to note that the lower parasitism rate during the VOC collection experiment may have made it difficult to detect the parasitism signal in VOCs associated with parasitised *B. brassicae* aphids, as density effects are important for aphid‐induced plant responses under dual herbivory (Ponzio *et al*., 2016; Kroes *et al*., 2017; Cascone *et al*., 2019). This may extend to the low percentage of parasitised aphids leading to a different signal than a higher percentage of parasitised aphids would. Hence, repeating the volatile collection experiment with a higher parasitism rate or longer collection time would be of significant value to pinpoint the difference in HIPVs caused by parasitised aphids.

The hyperparasitoid is not attracted to plants infested with (parasitised) *M. persicae* despite the presence of several known attractive VOCs in their headspace. Analysis of volatile compounds indicates that *beta*‐caryophyllene and methyl thiocyanate are mostly related to induction with (parasitised) *M. persicae*. *Beta‐*caryophyllene is known to be a plant‐produced attractant to parasitoid wasps after aphid induction (Vitiello *et al*., 2021). Methyl thiocyanate is a product derived from hydrolysed glucosinolate and has a strong biological activity against bacteria, increasing plant resistance against bacterial pathogens that invade wounded sites (Nagatoshi & Nakamura, 2009). The presence of volatile methyl thiocyanate indicates a strong activity of myrosinases and that parasitised *M. persicae* aphids cause more tissue damage during their feeding than unparasitised *M. persicae* aphids. Yet, the pathway parameters of aphid feeding (activity where most cell damage caused by a stylet happens) were not different. Besides the composition of the volatile blend and amount of specific compounds, also the total amount of VOCs is thought to play a role in guiding parasitoid foraging (Turlings & Erb, 2018; Lin *et al*., 2022) and can be potentially important for the host location of hyperparasitoids. Our volatile analyses were based on abundances obtained through an untargeted metabolomics workflow. Although this approach is powerful for detecting compound‐specific differences, it does not provide absolute quantification of emission rates. Hence, we cannot exclude the possibility that quantitative changes in total HIPV production contribute to hyperparasitoid preference.

SA ‐ and ET‐mediated responses were identified as induced hormonal pathways after induction with (parasitised) aphids. A homologous gene to *Arabidopsis thaliana PR2* was identified to be most strongly upregulated upon induction with parasitised *B. brassicae* in comparison with unparasitised *B. brassicae*. *PR2* is known to be involved in plant defences against fungal pathogens and is an ABA‐mediated repressor of callose depositions as the encoded *beta*‐1,3‐glucanase degrades callose (Oide *et al*., 2013). Its strong upregulation indicates that callose depositions, which are an effective defence mechanism against aphids, are repressed or even degraded (Oide *et al*., 2013; Will *et al*., 2013; Escudero‐Martinez *et al*., 2017). Phenotypic effects must be verified to confirm this, for example through aniline blue staining, which is often used for the localisation and quantification of callose depositions (Kim *et al*., 2020). In line with previous reports, we found that parasitised aphids exhibited similar stylet activities in the pathway and phloem as unparasitised aphids, although they interact more often with the xylem (Ramírez *et al*., 2006). Therefore, we do not consider it likely that the differences in plant responses to parasitised and unparasitised aphids in our study are the result of differences in feeding behaviour after parasitism.

Interestingly, *A. fuscicornis* preferred plants infested with unparasitised *B. brassicae* over uninfested control plants. This suggests that the presence of *B. brassicae* alone may already provide hyperparasitoids with cues that increase the likelihood of future host encounters. Similar patterns have been reported in caterpillar‐based systems, in which hyperparasitoids respond not only to HIPVs induced by parasitised hosts but also to those induced by unparasitised herbivores, which represent future opportunities for host exploitation (Poelman *et al*., 2012; Cusumano *et al*., 2019). Such behaviour may reflect a hierarchical foraging strategy, where hyperparasitoids first use more general herbivore‐induced cues to locate potential host habitats and then rely on more specific cues to detect parasitised hosts (Bourne *et al*., 2024). From an applied perspective, our results further suggest that hyperparasitoids may disrupt aphid biocontrol more strongly in *B. brassicae*‐dominated systems, potentially even causing local extinctions (Frago, 2016; Kehoe *et al*., 2021). Furthermore, knowledge of when and how hyperparasitoids respond to plant cues can help identify volatile markers that reliably signal their activity. These could be used to design bait‐and‐trap systems for early detection or suppression of hyperparasitoids. As a next step, candidate volatiles from the *B. brassicae–D. rapae* system (e.g. ((*E*)‐DMNT)) could be tested in targeted experiments to reveal their potential in push–pull strategies targeting aphid hyperparasitoids (Cusumano *et al*., 2020). Investigating whether hyperparasitised aphids elicit distinct volatile and transcriptomic responses compared to parasitised aphids would deepen this knowledge further to disentangle the role of (hyper)parasitised hosts in shaping plant–insect interactions.

To conclude, our study demonstrates that an aphid hyperparasitoid uses the phenotypic alterations of a plant colonised by parasitised aphids to locate its parasitoid host. *A. fuscicornis* has specialised to use cues induced by its most prevalent aphid–parasitoid association in its food web but does not exploit similar cues induced by its less common host. Our findings reveal that this phenomenon is aphid species‐specific and emphasise the intricacy of multitrophic interaction networks and the importance of considering the dynamic and context‐dependent nature of plant–insect interactions (Cuny *et al*., 2021; Mertens *et al*., 2021). Future studies could explore whether alterations in the aphid microbiome and secreted proteins after parasitism influence induced plant responses and, subsequent trophic interactions. This will further improve our understanding of the chemical‐ and microbial ecology mediating species interactions up to the fourth trophic level.

## Competing interests

None declared.

## Author contributions

MEB and EHP conceived and designed the research. MEB, AV, GAC, LM and BTW conducted the experiments. MEB, GAC, AV, BTW and KJK analysed the data. MEB and EHP wrote the first version of the manuscript. All authors contributed to data interpretation and critical revisions of the manuscript.

## Disclaimer

The New Phytologist Foundation remains neutral with regard to jurisdictional claims in maps and in any institutional affiliations.

## Supporting information


**Fig. S1** Parasitism rates in different experiments with parasitised aphids.
**Fig. S2** Analysis of separate volatile compounds and the volatile profiles of *Brassica oleracea* ‘Kimmeridge’ plants induced with (parasitised) aphids for 96 h.
**Fig. S3** *Brassica oleracea* ‘Kimmeridge’ plant responses in defence‐related pathways for the comparisons between plants induced with parasitised and unparasitised aphids of the same species, 96 h after induction with respective treatment.
**Methods S1** Y‐tube olfactometer bioassays.
**Methods S2** Collection and analysis of volatile organic compounds.
**Methods S3** Transcriptomics of (parasitised) aphid‐induced leaf samples.
**Table S1** RNA‐seq sequencing depth, mapping average number of mummies per sample.
**Table S2** Outcome of multivariate PERMANOVA models for volatile blends and *Brassica oleracea* ‘Kimmeridge’ plant transcriptome after induction with (parasitised) aphids.
**Table S3** *Brassica oleracea* ‘Kimmeridge’ plant VOC analysis after induction with (parasitised) aphids, using a DESeq2 approach.
**Table S4** VOCs in the headspace of (parasitised) aphid‐induced *Brassica oleracea* ‘Kimmeridge’ plants or uninduced control plants.
**Table S5** Outcome of pairwise PERMANOVA over all treatments in the *Brassica oleracea* ‘Kimmeridge’ plant transcriptome dataset, where plants were induced with (parasitised) aphids or undamaged control, to compare the expression between individual treatments.
**Table S6** Top and bottom 15 loadings of PC1 of the *Brassica oleracea* ‘Kimmeridge’ RNAseq dataset (Fig. 4b).
**Table S7** Top and bottom 15 loadings of PC2 of the *Brassica oleracea* ‘Kimmeridge’ RNAseq dataset (Fig. 4b).
**Table S8** Differentially expressed genes (DEGs) in *Brassica oleracea* ‘Kimmeridge’ with log_2_FC > 1 for the Bb.par vs Bb comparison.
**Table S9** Differentially expressed genes (DEGs) in *Brassica oleracea* ‘Kimmeridge’ with log_2_FC > 1 for the Mp.par vs Mp comparison.
**Table S10** Extended EPG table.
Please note: Wiley is not responsible for the content or functionality of any Supporting Information supplied by the authors. Any queries (other than missing material) should be directed to the *New Phytologist* Central Office.

## Data Availability

Raw sequencing data are available from NCBI (BioProject accession no.: PRJNA989068; https://www.ncbi.nlm.nih.gov/bioproject/PRJNA989068). R‐code is publicly available at: doi: 10.6084/m9.figshare.30265930.v1. Other datasets are available on Figshare. Processed read files of RNA‐sequencing dataset (doi: 10.6084/m9.figshare.29149217.v1) Hyperparasitoid choice data. (doi: 10.6084/m9.figshare.26076901.v1), EPG data (doi: 10.6084/m9.figshare.26076904.v1), Annotated EPG files (doi: 10.6084/m9.figshare.29517524.v1). VOC data (doi: 10.6084/m9.figshare.26076880.v1).
