# Supplementary material for: Aphid parasitism alters induced plant responses allowing a hyperparasitoid to locate its hidden parasitoid host
Source: New Phytol. 2025 Dec 15;249(4):2055–70. doi: 10.1111/nph.70774 (PMC12825394; doi:10.1111/nph.70774)
Supplement: Supplementary file 1 — Fig. S1 Parasitism rates in different experiments with parasitised aphids. Fig. S2 Analysis of separate volatile compounds and the volatile profiles of Brassica oleracea ‘Kimmeridge’ plants induced with (parasitised) aphids for 96 h. Fig. S3 Brassica oleracea ‘Kimmeridge’ plant responses in defence‐related pathways for the comparisons between plants induced with parasitised and unparasitised aphids of the same species, 96 h after induction with respective treatment. Methods S1 Y‐tube olfactometer bioassays. Methods S2 Collection and analysis of volatile organic compounds. Methods S3 Transcriptomics of (parasitised) aphid‐induced leaf samples. Table S1 RNA‐seq sequencing depth, mapping average number of mummies per sample. Table S2 Outcome of multivariate PERMANOVA models for volatile blends and Brassica oleracea ‘Kimmeridge’ plant transcriptome after induction with (parasitised) aphids. Table S3 Brassica oleracea ‘Kimmeridge’ plant VOC analysis after induction with (parasitised) aphids, using a DESeq2 approach. Table S4 VOCs in the headspace of (parasitised) aphid‐induced Brassica oleracea ‘Kimmeridge’ plants or uninduced control plants. Table S5 Outcome of pairwise PERMANOVA over all treatments in the Brassica oleracea ‘Kimmeridge’ plant transcriptome dataset, where plants were induced with (parasitised) aphids or undamaged control, to compare the expression between individual treatments. Table S6 Top and bottom 15 loadings of PC1 of the Brassica oleracea ‘Kimmeridge’ RNAseq dataset (Fig. 4b). Table S7 Top and bottom 15 loadings of PC2 of the Brassica oleracea ‘Kimmeridge’ RNAseq dataset (Fig. 4b). Table S8 Differentially expressed genes (DEGs) in Brassica oleracea ‘Kimmeridge’ with log2FC > 1 for the Bb.par vs Bb comparison. Table S9 Differentially expressed genes (DEGs) in Brassica oleracea ‘Kimmeridge’ with log2FC > 1 for the Mp.par vs Mp comparison. Table S10 Extended EPG table. Please note: Wiley is not responsible for the content or functionality of any Suppo [file NPH-249-2055-s001.pdf]

***New Phytologist* Supporting Information**

Article title: Aphid parasitism alters induced plant responses allowing a hyperparasitoid to locate its hidden parasitoid host

Authors: Mitchel E. Bourne[1,2*], Alessia Vitiello[1#], Gabriel Charvalakis[1&], Leandra Meerkerk[1], Berhane T. Weldegergis[1], Karen J. Kloth[1] and Erik H. Poelman[1*]

[1] Laboratory of Entomology, Wageningen University & Research, Droevendaalsesteeg 1, 6708 PB Wageningen, The Netherlands.

[2] National Centre for Vector Entomology, Institute of Parasitology, Vetsuisse and Medical Faculty, One Health Institute, University of Zürich, Winterthurerstrasse 266A, 8057 Zürich, Switzerland

*Corresponding authors: mitchel.bourne@uzh.ch and erik.poelman@wur.nl

Article acceptance date: 31 October 2025

The following supporting information is available for this article:

Figures S1 to S3

Tables S1 to S9

Methods S1 to S3

SI References: 16 references

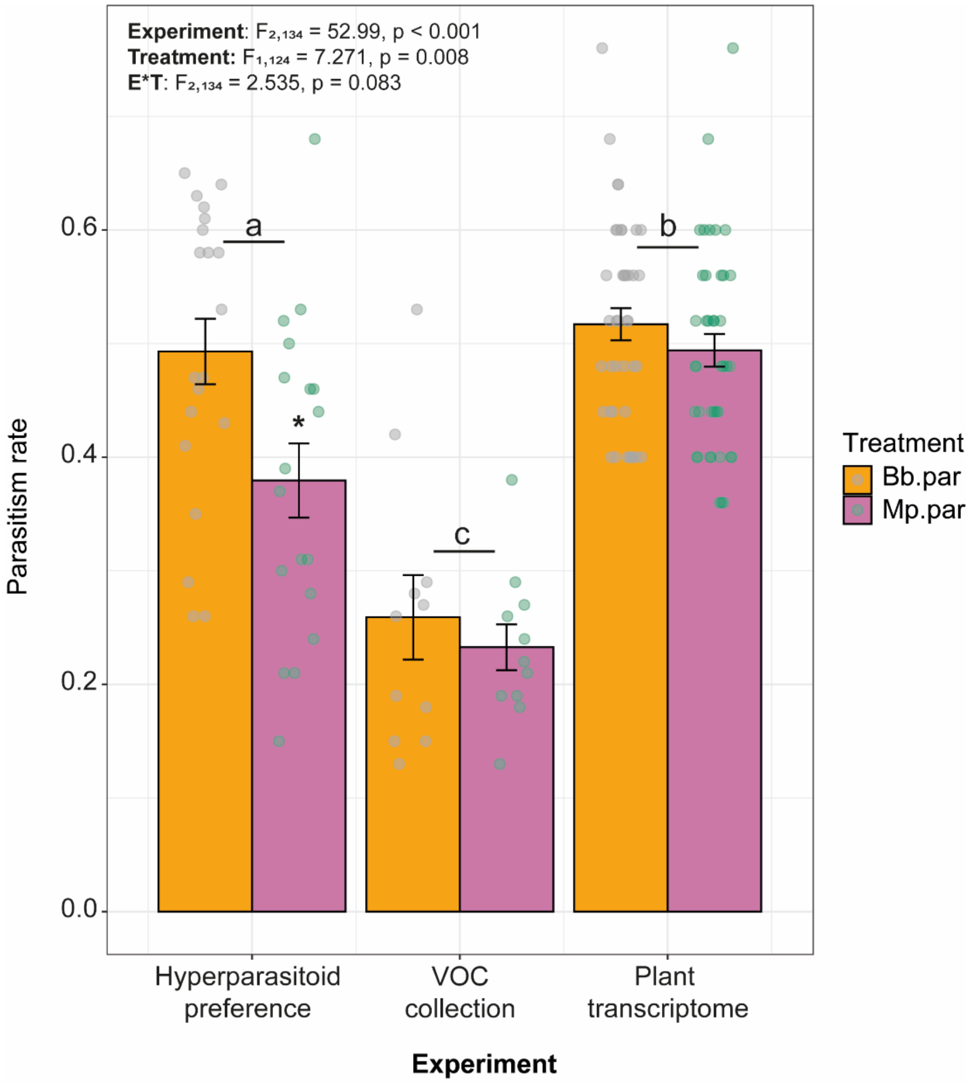

Fig S1: Parasitism rates in different experiments with parasitised aphids. The parasitism rate was calculated as the number of mummies found on the plant (a week after the experiment ended) divided by the total number of aphids that the plant was induced with. Letters indicate differences between the parasitism rate of different experiments ANOVA with Tukey's HSD for pairwise comparisons ($p < 0.05$). Asterisks indicate differences between treatments within the same experiment (T-test: $p < 0.05$). Both aphid species, where Bb represents *B. brassicae* and Mp represents *M. persicae,* were parasitised by their specialist parasitoid. Bb.par = *B. brassicae* parasitised by parasitoid *D. rapae.* Mp.par = *M. persicae* parasitised by its parasitoid *A. colemani*. Error bars represent the standard error of the mean.

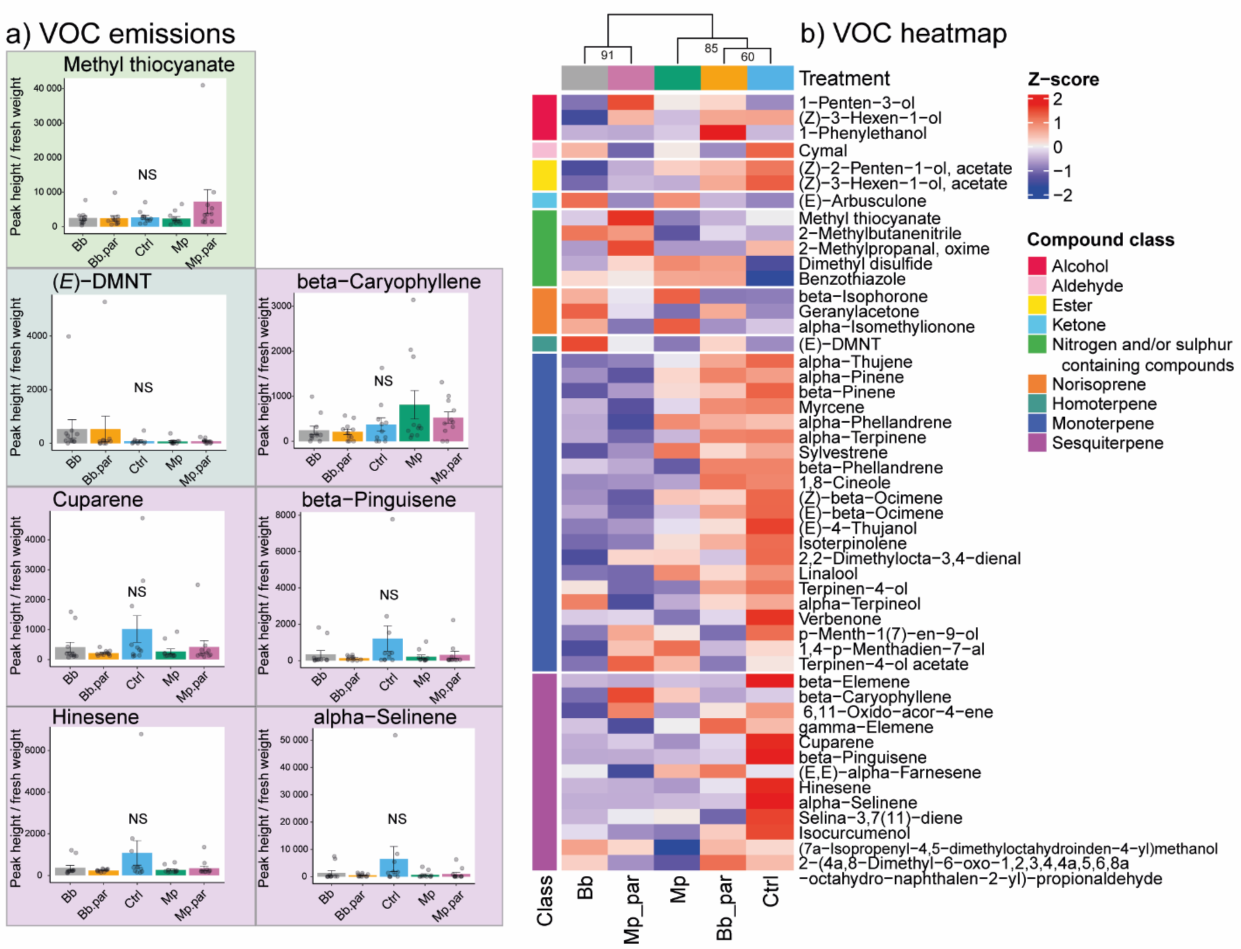

Fig S2: Analysis of separate volatile compounds and the volatile profiles of *B. oleracea* "Kimmeridge" plants induced with (parasitised) aphids for 96 hours. a) VOC emissions of selected compounds (peak height/ plant fresh weight). Bars indicate the average value per plant fresh weight +/- the standard error of the mean. b) Heatmap of all volatile compounds in the blends, peak heights per compound were centred and scaled. Clustering based on Euclidian distance with complete linkage. Numbers in the cluster dendrogram indicate the approximately unbiased (AU) p-value. (*E*)-DMNT is an abbreviation for the compound (*E*)-4,8-dimethyl-1,3,7-nonatriene. Both aphid species, where Bb represents *B. brassicae* and Mp represents *M. persicae,* were parasitised by their specialist parasitoid. Bb.par = *B. brassicae* parasitised by parasitoid *D. rapae.* Mp.par = *M. persicae* parasitised by its parasitoid *A. colemani*. Letters in (a) indicate significant differences with $p < 0.05$ for Dunn's test with a Hochberg correction. NS = no significant differences amongst treatments. n = 11 biological replicates per treatment, each replicate consisting of volatiles collected from a single plant..

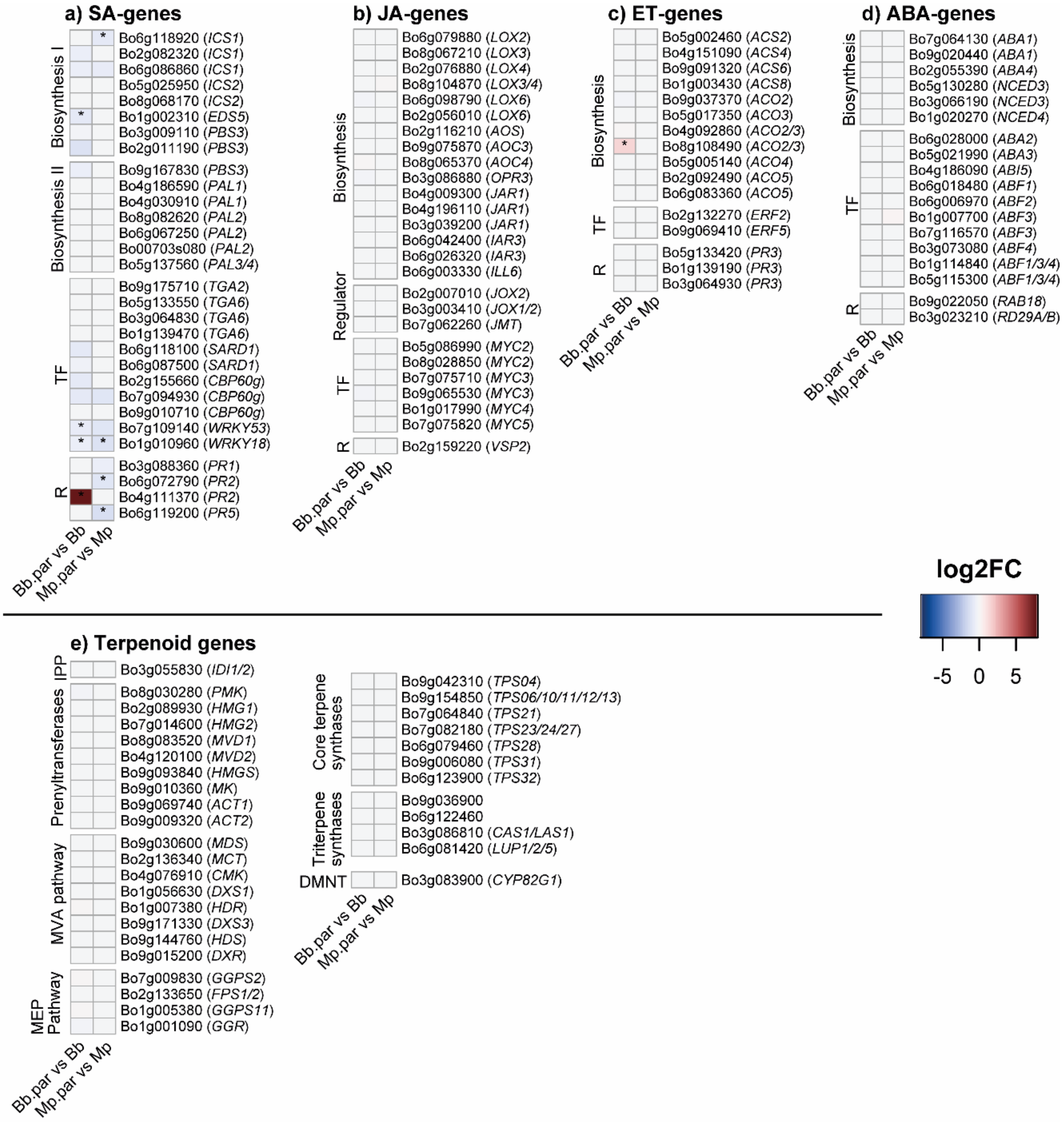

Figure S3: *B. oleracea* "Kimmeridge" plant responses in defence-related pathways for the comparisons between plants induced with parasitised and unparasitised aphids of the same species, 96 hours after induction with respective treatment. Both aphid species, where Bb represents *B. brassicae* and Mp represents *M. persicae,* were parasitised by their specialist parasitoid. Bb.par = *B. brassicae* parasitised by parasitoid *D. rapae.* Mp.par = *M. persicae* parasitised by its parasitoid *A. colemani*. a) Salicylic acid (SA) pathway. b) Jasmonic acid (JA) pathway. c) Ethylene pathway (ET). d) Abscisic acid pathway (ABA) e) Genes related to terpenoid biosynthesis. Asterisks indicate DEGs compared to uninduced control plants with $p < 0.01$ and absolute log2FC larger than 0.5, calculated with DESeq2. Used abbreviations: IPP = isopentenyl diphosphate; MVA = mevalonate; MEP = methylerythritol phosphate; DMNT = 4,8-dimethyl-1,3,7-nonatriene. For these contrasts, treatments were analysed in subsets (Bb.par vs Bb; Mp.par vs Mp), which involved re-estimating size factors, dispersion, and the apeglm prior.

Table S1: RNA-seq sequencing depth, mapping average number of mummies per sample. Both aphid species, where Bb represents *B. brassicae* and Mp represents *M. persicae,* were parasitised by their specialist parasitoid. Bb.par = *B. brassicae* parasitised by parasitoid *D. rapae.* Mp.par = *M. persicae* parasitised by its parasitoid *A. colemani*. n = 5 biological replicates per treatment, each replicate consisting of pooled leaf tissue from 8 *B. oleracea* “Kimmeridge” plants.

ID	Technology	Run type	Sample	Treatment	Untrimmed reads (mil)	Trimmed reads (mil)	GC content (%)	Alignment percentage	Aligned reads (mil)	Average Number of mummies
A1	dnbSEQ	150bp PE	Ctrl_1	Ctrl	34.4	34.4	47.6	91.20%	31.4	0.0
A2	dnbSEQ	150bp PE	Ctrl_2	Ctrl	34.2	34.2	47.9	91.50%	31.3	0.0
A3	dnbSEQ	150bp PE	Ctrl_3	Ctrl	34.4	34.4	47.6	91.10%	31.3	0.0
A4	dnbSEQ	150bp PE	Ctrl_4	Ctrl	33.8	33.8	47.8	91.30%	30.9	0.0
A5	dnbSEQ	150bp PE	Ctrl_5	Ctrl	33.8	33.8	48.0	91.20%	30.8	0.0
B1	dnbSEQ	150bp PE	Bb_1	Bb	34.2	34.2	47.5	90.00%	30.8	0.0
B2	dnbSEQ	150bp PE	Bb_2	Bb	34.3	34.3	47.6	90.50%	31.0	0.0
B3	dnbSEQ	150bp PE	Bb_3	Bb	34.6	34.6	47.3	90.30%	31.3	0.0
B4	dnbSEQ	150bp PE	Bb_4	Bb	33.3	33.3	47.6	90.70%	30.2	0.0
B5	dnbSEQ	150bp PE	Bb_5	Bb	31.8	31.8	47.6	90.70%	28.8	0.0
M1	dnbSEQ	150bp PE	Mp_1	Mp	34.4	34.4	47.4	90.50%	31.2	0.0
M2	dnbSEQ	150bp PE	Mp_2	Mp	34.5	34.5	47.6	90.90%	31.4	0.0
M3	dnbSEQ	150bp PE	Mp_3	Mp	33.8	33.8	47.7	89.80%	30.4	0.0
M4	dnbSEQ	150bp PE	Mp_4	Mp	34.4	34.4	47.7	90.70%	31.2	0.0
M5	dnbSEQ	150bp PE	Mp_5	Mp	33.5	33.5	47.7	90.90%	30.4	0.0
X1	dnbSEQ	150bp PE	Mp.par_1	Mp.par	33.0	33.0	47.7	91.00%	30.1	12.4
X2	dnbSEQ	150bp PE	Mp.par_2	Mp.par	33.6	33.6	47.8	90.70%	30.4	12.5
X3	dnbSEQ	150bp PE	Mp.par_3	Mp.par	33.7	33.7	47.8	90.90%	30.6	12.3
X4	dnbSEQ	150bp PE	Mp.par_4	Mp.par	33.8	33.8	47.9	91.30%	30.9	12.4
X5	dnbSEQ	150bp PE	Mp.par_5	Mp.par	33.7	33.7	47.8	90.10%	30.3	12.3
Z1	dnbSEQ	150bp PE	Bb.par_1	Bb.par	33.7	33.7	47.5	90.80%	30.6	12.9
Z2	dnbSEQ	150bp PE	Bb.par_2	Bb.par	33.5	33.5	47.6	90.70%	30.3	13.0
Z3	dnbSEQ	150bp PE	Bb.par_3	Bb.par	33.5	33.5	47.8	91.10%	30.5	13.1
Z4	dnbSEQ	150bp PE	Bb.par_4	Bb.par	33.2	33.2	47.5	90.40%	30.0	12.9
Z5	dnbSEQ	150bp PE	Bb.par_5	Bb.par	33.0	33.0	47.7	90.50%	29.9	12.8

Table S2: Outcome of multivariate PERMANOVA models for volatile blends and *B. oleracea* “Kimmeridge” plant transcriptome after induction with (parasitised) aphids. a) Uncorrected VOC emissions. b) plant transcriptome. Both aphid species, where Bb represents *B. brassicae* and Mp represents *M. persicae,* were parasitised by their specialist parasitoid. Bb.par = *B. brassicae* parasitised by parasitoid *D. rapae.* Mp.par = *M. persicae* parasitised by its parasitoid *A. colemani*. Used abbreviations: Df = degrees of freedom, SSQ = sum of squares. Inducer identity represents the aphid species (Mp or Bb), parasitism rate represents a binary factor (presence/absence of parasitised aphids), and parasitism rate represents a continuous factor with the number of mummies found on the plant compared to the number of aphids it was induced with.

**a) VOC dataset (peak height per aboveground plant fresh weight)**								
	**Dataset**	**Model**	**Factor**	**Df**	**SSQ**	**R^2**	**F**	**p**
**i)**	Full	~ Treatment	Treatment	4	0.4556	0.061	0.82	0.641
**ii)**	Full minus Ctrl	~ Inducer_identity * Parasitism	Inducer_identity	1	0.1549	0.02626	1.1143	0.311
			Parasitism	1	0.1020	0.01730	0.7338	0.539
			Inducer_identity*parasitism	1	0.0804	0.01363	0.5784	0.677
**iii)**	Full minus Ctrl	~ Inducer_identity * Parasitism_rate	Inducer_identity	1	0.1549	0.02626	1.1515	0.304
			Parasitism_rate	1	0.2491	0.04224	1.8517	**0.122**
			Inducer_identity*parasitism rate	1	0.1126	0.01910	0.8374	0.489
**b) Transcriptome dataset**								
	Dataset	**Model**	**Factor**	**Df**	**SSQ**	**R^2**	**F**	**p**
**i)**	Full	~ Treatment	Treatment	4	0.016914	0.84473	27.202	**<0.001**
**ii)**	Full minus Ctrl	~ Inducer_identity * Parasitism	Inducer_identity	1	0.005762	0.57071	34.0287	**<0.001**
			Parasitism	1	0.00159	0.15747	9.3893	**0.006**
			Inducer_identity*parasitism	1	0.000035	0.00347	0.2068	0.886
**iii)**	Full minus Ctrl	~ Inducer_identity * Parasitism_rate	Inducer_identity	1	0.005762	0.57071	34.0717	**<0.001**
			Parasitism_rate	1	0.001586	0.15707	9.3774	**0.002**
			Inducer_identity*parasitism rate	1	4.24E-05	0.0042	0.251	0.852

Table S3: *B. oleracea* "Kimmeridge" plant VOC analysis after induction with (parasitised) aphids, using a DESeq2 approach. We used the rounded peak height/plant fresh weight per compound, corrected for SizeFactor and calculated differential VOCs. We used a model with a combined factor for all treatments, which was relevelled to control treatment as baseline, and VOCs were classified as differential if they were different from the control with a false discovery rate lower than 0.05. Compounds with at least one significant difference are represented in the table. Both aphid species, where Bb represents *B. brassicae* and Mp represents *M. persicae,* were parasitised by their specialist parasitoid. Bb.par = *B. brassicae* parasitised by parasitoid *D. rapae.* Mp.par = *M. persicae* parasitised by its parasitoid *A. colemani*. (*E*)-DMNT is an abbreviation for the compound (*E*)-4,8-dimethyl-1,3,7-nonatriene. Padj represents the adjusted p-value of the analysis and log2FC the log2 foldchange. Asterisks indicate significant differences between control and respective treatment.

Compound	Bb	Bb.par	Mp	Mp.par
Methyl thiocyanate	Padj = 0.562<br>log2FC = 0.006	Padj = 0.989<br>log2FC = 0.001	Padj = 0.967<br>log2FC = -0.002	**Padj = 0.008***<br>**log2FC = 1.280**
(*E*)-DMNT	Padj = 0.326354<br>log2FC = 0.009	**Padj = 0.044***<br>**log2FC = 0.036**	Padj = 0.913<br>log2FC = 0.011	Padj = 0.606<br>log2FC = 0.073
beta-Caryophyllene	Padj = 0.828769<br>log2FC = -0.002	Padj = 0.989<br>log2FC = -0.009	Padj = 0.635<br>log2FC = 0.047	Padj = 0.404<br>log2FC = 0.168
Cuparene	Padj = 0.326354<br>log2FC = -0.019	**Padj = 0.013***<br>**log2FC = -1.387**	Padj = 0.361<br>log2FC = -0.190	Padj = 0.462<br>log2FC = -0.205
beta-Pinguisene	Padj = 0.326354<br>log2FC = -0.012	**Padj = 0.032***<br>**log2FC = -0.053**	Padj = 0.493<br>log2FC = -0.074	Padj = 0.400<br>log2FC = -0.198
Hinesene	Padj = 0.326354<br>log2FC = -0.025	**Padj = 0.011***<br>**log2FC = -1.253**	Padj = 0.299<br>log2FC = -0.687	Padj = 0.338523<br>log2FC = -0.391
alpha-Selinene	Padj = 0.326354<br>log2FC = -0.009	**Padj = 0.037***<br>**log2FC = -0.040**	Padj = 0.361<br>log2FC = -0.065	Padj = 0.32167<br>log2FC = -0.231

Table S4: VOCs in the headspace of (parasitised) aphid-induced *B. oleracea* "Kimmeridge" plants or uninduced control plants. Values represent size-factor-normalised raw peak heights (mean (± SE)). These are the values that underpin the DESeq2 analysis in Table S1. Both aphid species, where Bb represents *B. brassicae* and Mp represents *M. persicae,* were parasitised by their specialist parasitoid. Bb.par = *B. brassicae* parasitised by parasitoid *D. rapae.* Mp.par = *M. persicae* parasitised by its parasitoid *A. colemani*. (*E*)-DMNT is an abbreviation for the compound (*E*)-4,8-dimethyl-1,3,7-nonatriene.

Compound	CTRL	Bb	Bb.par	Mp	Mp.par
**ALCOHOLS**					
1-Penten-3-ol	5959.9 (± 2085.4)	7171.4 (± 3453.6)	5928.2 (± 2773.5)	5036.5 (± 958.9)	9097.7 (± 2268.7)
(*Z*)-3-Hexen-1-ol	48688.0 (± 19154.3)	27790.8 (± 17735.2)	66131.9 (± 32766.9)	32279.0 (± 8166.1)	51867.7 (± 17884.0)
1-Phenylethanol	2644.9 (± 818.2)	4365.4 (± 3015.0)	2987.1 (± 552.4)	3160.2 (± 1306.8)	2397.9 (± 859.5)
**ALDEHYDE**					
Cymal	149.2 (± 28.7)	185.8 (± 52.8)	127.3 (± 38.0)	133.8 (± 27.0)	185.1 (± 82.2)
**ESTERS**					
(*Z*)-2-Penten-1-ol, acetate	981.1 (± 392.2)	672.0 (± 313.7)	1206.2 (± 638.1)	678.8 (± 199.0)	612.0 (± 170.2)
(*Z*)-3-Hexen-1-ol, acetate	47952.1 (± 16129.2)	30222.0 (± 14123.6)	56796.8 (± 25704.7)	40125.9 (± 11786.7)	41766.1 (± 12728.1)
**KETONE**					
(*E*)-Arbusculone	444.9 (± 105.2)	535.2 (± 118.7)	484.6 (± 119.1)	639.5 (± 177.9)	755.9 (± 221.1)
Methyl thiocyanate	2005.6 (± 249.5)	2353.1 (± 487.5)	2018.6 (± 653.8)	1983.4 (± 314.0)	5654.2 (± 1779.7)
2-Methylbutanenitrile	2013.5 (± 692.9)	2101.5 (± 645.8)	3440.7 (± 1621.4)	1958.9 (± 756.6)	2706.1 (± 722.8)
2-Methylpropanal, oxime	302.0 (± 222.2)	88.0 (± 43.9)	20.5 (± 14.1)	97.5 (± 65.6)	188.6 (± 91.0)
Dimethyl disulfide	6150.7 (± 2471.7)	9120.7 (± 3318.7)	5249.6 (± 655.0)	9515.6 (± 2175.0)	12140.1 (± 6600.1)
Benzothiazole	17713.8 (± 2955.8)	22333.8 (± 4189.6)	18865.4 (± 2597.0)	24050.0 (± 4464.3)	27425.7 (± 4235.1)
**NORISOPRENES**					
beta-Isophorone	1590.2 (± 356.6)	3108.7 (± 648.6)	2598.2 (± 726.7)	3018.6 (± 576.4)	3184.3 (± 721.2)
Geranylacetone	1828.8 (± 302.1)	2789.6 (± 619.3)	2580.1 (± 362.1)	2511.3 (± 519.5)	2909.5 (± 463.0)
alpha-Isomethylionone	1578.9 (± 388.9)	1888.6 (± 398.6)	1336.5 (± 216.8)	1669.3 (± 194.1)	1880.1 (± 385.1)
**HOMOTERPENE**					
(*E*)-DMNT	59.0 (± 19.5)	292.4 (± 105.2)	452.5 (± 402.1)	78.4 (± 29.2)	98.5 (± 33.5)
**MONOTERPENES**					
alpha-Thujene	48447.1 (± 9952.0)	37379.0 (± 9951.3)	44652.8 (± 7043.1)	29973.2 (± 8240.3)	25740.0 (± 5842.6)
alpha-Pinene	27577.8	25121.7	27370.4	22729.2	14758.2

	(± 3872.7)	(± 5440.8)	(± 4849.9)	(± 4741.1)	(± 2600.4)
beta-Pinene	101411.9 (± 13479.1)	94816.4 (± 13243.1)	99538.6 (± 13800.0)	73301.7 (± 15974.4)	69813.8 (± 11948.0)
Myrcene	48259.5 (± 7991.1)	39148.9 (± 7754.7)	45310.7 (± 4970.1)	38857.5 (± 4659.0)	33973.8 (± 4878.8)
alpha-Phellandrene	1184.4 (± 229.1)	939.6 (± 185.0)	955.9 (± 126.0)	758.5 (± 154.8)	611.1 (± 100.8)
alpha-Terpinene	1713.1 (± 365.2)	1331.6 (± 353.1)	1532.2 (± 248.0)	1078.7 (± 305.0)	947.5 (± 213.2)
Sylvestrene	63798.4 (± 9520.6)	55004.3 (± 10120.3)	60578.3 (± 6869.7)	48705.8 (± 9145.7)	47418.6 (± 7592.1)
beta-Phellandrene	1121.4 (± 504.9)	826.8 (± 361.3)	799.8 (± 263.9)	602.9 (± 316.4)	471.4 (± 120.3)
1,8-Cineole	13543.8 (± 3165.0)	9594.7 (± 2734.0)	11102.3 (± 1923.3)	8677.1 (± 2422.0)	6821.9 (± 1470.6)
(*Z*)-beta-Ocimene	128.3 (± 26.1)	67.2 (± 20.3)	90.9 (± 14.3)	64.5 (± 17.3)	59.7 (± 18.5)
(*E*)-beta-Ocimene	7112.7 (± 1869.6)	5131.9 (± 1492.9)	4889.7 (± 731.8)	4014.0 (± 1141.8)	3157.8 (± 585.4)
(*E*)-4-Thujanol	2222.2 (± 369.7)	1845.5 (± 303.2)	1967.8 (± 272.5)	1734.1 (± 271.9)	1583.0 (± 220.9)
Isoterpinolene	1048.4 (± 236.7)	772.7 (± 188.2)	816.7 (± 118.3)	607.7 (± 138.9)	532.0 (± 99.2)
2,2-Dimethylocta-3,4-dienal	1271.8 (± 126.7)	1741.0 (± 247.6)	1394.5 (± 142.3)	1617.7 (± 262.0)	1887.2 (± 238.7)
Linalool	295.2 (± 55.2)	301.2 (± 65.1)	274.4 (± 35.1)	260.7 (± 34.9)	246.8 (± 30.5)
Terpinen-4-ol	747.6 (± 94.7)	783.5 (± 100.2)	690.7 (± 60.4)	570.7 (± 57.9)	601.8 (± 41.2)
alpha-Terpineol	658.6 (± 151.5)	941.7 (± 195.8)	937.5 (± 250.5)	673.9 (± 149.7)	568.7 (± 127.6)
Verbenone	366.4 (± 52.6)	457.9 (± 71.3)	417.3 (± 35.6)	485.4 (± 87.7)	529.9 (± 61.0)
p-Menth-1(7)-en-9-ol	457.4 (± 106.6)	522.4 (± 82.1)	381.9 (± 63.2)	529.7 (± 72.9)	583.4 (± 126.4)
1,4-p-Menthadien-7-al	577.4 (± 115.8)	540.0 (± 74.1)	622.1 (± 98.3)	936.7 (± 230.9)	868.9 (± 169.1)
Terpinen-4-ol-acetate	314.4 (± 79.3)	423.5 (± 124.9)	248.9 (± 59.5)	329.2 (± 82.3)	414.4 (± 123.7)
**SESQUITERPENES**					
beta-Elemene	6093.6 (± 2444.8)	2187.5 (± 1231.5)	1069.2 (± 393.7)	1987.1 (± 1148.1)	3210.0 (± 2942.1)
beta-Caryophyllene	250.1 (± 73.3)	189.1 (± 44.0)	182.6 (± 56.4)	592.7 (± 220.0)	582.9 (± 165.4)
6,11-Oxido-acor-4-ene	1968.6 (± 160.7)	2134.6 (± 251.3)	2192.3 (± 465.9)	2380.2 (± 401.7)	2643.9 (± 541.2)
gamma-Elemene	2083.2 (± 538.7)	3945.4 (± 1347.7)	2245.2 (± 370.6)	2283.1 (± 484.6)	3237.6 (± 972.8)
Cuparene	634.9 (± 205.8)	298.4 (± 66.1)	186.8 (± 24.1)	268.5 (± 67.6)	419.2 (± 173.7)
beta-Pinguisene	652.2 (± 251.6)	188.9 (± 85.1)	111.6 (± 29.5)	201.7 (± 78.4)	276.9 (± 166.0)
(*E,E*)-alpha-Farnesene	602.0 (± 308.2)	1180.5 (± 548.7)	345.9 (± 86.3)	480.2 (± 153.5)	1072.3 (± 500.1)

Hinesene	595.9 (± 193.6)	320.0 (± 57.8)	207.4 (± 15.6)	271.5 (± 48.0)	357.3 (± 86.2)
alpha-Selinene	3008.8 (± 1459.4)	668.9 (± 378.4)	416.9 (± 127.3)	652.9 (± 283.3)	822.9 (± 502.4)
Selina-3,7(11)-diene	203.4 (± 78.2)	61.2 (± 24.4)	28.0 (± 12.0)	74.1 (± 24.1)	90.9 (± 43.3)
Isocurcumenol	149.4 (± 37.8)	165.6 (± 50.0)	140.6 (± 48.6)	135.6 (± 36.1)	174.7 (± 71.0)
(7a-Isopropenyl-4,5-dimethyloctahydroinden-4-yl) methanol	32572.7 (± 4836.3)	43208.6 (± 12358.1)	31517.7 (± 7166.4)	33790.1 (± 5310.3)	40945.1 (± 10646.2)
2-(4a,8-Dimethyl-6-oxo-1,2,3,4,4a,5,6,8a-octahydro-naphthalen-2-yl)-propionaldehyde	459.8 (± 89.0)	605.7 (± 190.4)	442.7 (± 127.4)	443.9 (± 84.7)	570.3 (± 178.7)

Data represents the mean (± standard error)

Table S5: Outcome of pairwise PERMANOVA over all treatments in the *B. oleracea* "Kimmeridge" plant transcriptome dataset, where plants were induced with (parasitised) aphids or undamaged control, to compare the expression between individual treatments. Both aphid species, where Bb represents *B. brassicae* and Mp represents *M. persicae,* were parasitised by their specialist parasitoid. Bb.par = *B. brassicae* parasitised by parasitoid *D. rapae.* Mp.par = *M. persicae* parasitised by its parasitoid *A. colemani*. Used abbreviations: Df = degrees of freedom, SSQ = sum of squares.

	**Ctrl**	**Bb**	**Bb.par**	**Mp**	**Mp.par**
**Ctrl**	x	x	x	x	x
**Bb**	$F_{1,8}$ = 90.405, **p = 0.008**	x	x	x	x
**Bb.par**	$F_{1,8}$ = 71,873, **p = 0.007**	$F_{1,8}$ = 3.1082, **p = 0.038**	x	x	x
**Mp**	$F_{1,8}$ = 33.329, **p = 0.014**	$F_{1,8}$ = 14.256, **p = 0.010**	$F_{1,8}$ = 7.9189, **p = 0.007**	x	x
**Mp.par**	$F_{1,8}$ = 14.269, **p = 0.004**	$F_{1,8}$ = 31.473, **p = 0.012**	$F_{1,8}$ = 20.192, **p = 0.011**	$F_{1,8}$ = 4.69, **p = 0.028**	x

Table S6: Top and bottom 15 loadings of PC1 of the *B. oleracea* "Kimmeridge" RNAseq dataset (Fig 4b). *Arabidopsis* orthologs of genes were identified with PLAZA 4.5 and their functional characterisation was done with TAIR and ENSEMBL databases. Genes with asterisks were characterised based on protein structure with the UNIPROT database. Used abbreviations: BOL = *B. oleracea*, AT = *A. thaliana*.

PC	Rank	BOL gene	Loading score	AT ortholog (Best hit)	AT gene name	Description/function	Ortholology evidence[1]
1	Top 1	Bo3g024590	0.075	AT2G43590		PR-3 like gene that is induced by pathogen infection; Endochitinase*	1, 2, 3
1	Top 2	Bo3g055940	0.071	N/A		Uncharacterised protein*	N/A
1	Top 3	Bo6g083570	0.070	N/A		Extensin_2 domain-containing protein*	N/A
1	Top 4	Bo3g058570	0.069	AT3G04720	PR4	PATHOGENESIS-RELATED 4: Encodes a protein similar to the antifungal chitin-binding protein hevein from rubber tree latex.	1, 2, 3
1	Top 5	Bo01002s040	0.068	AT5G22570	WRKY38	WRKY DNA-BINDING PROTEIN 38: Member of WRKY Transcription Factor; Group III	1, 2, 3
1	Top 6	Bo5g027380	0.066	AT1G19320		Pathogenesis-related thaumatin superfamily protein*	1, 3
1	Top 7	Bo6g120910	0.064	N/A		Extensin domain-containing protein*	N/A
1	Top 8	Bo4g021480	0.063	AT2G44220	DUF239	Domain of unknown function 239 gene: Conserved Neprosin-like catalytic domain; possibly involved in seed development	1, 3
1	Top 9	Bo4g020920	0.063	AT2G43570	CHI	Endochitinase*	1, 2, 3, 4
1	Top 10	Bo5g004490	0.062	AT1G04350		1-aminocyclopropane-1-carboxylate oxidase (ACC) homolog 6*	1, 3, 4
1	Top 11	Bo2g151530	0.061	AT5G49360	BXL1	BETA-XYLOSIDASE 1: Bifunctional *beta*-D-xylosidase/*alpha*-L-arabinofuranosidase required for pectic arabinan modification	1, 2, 3, 4
1	Top 12	Bo6g119200	0.060	AT1G75050		Pathogenesis-related thaumatin superfamily protein*	1, 3
1	Top 13	Bo3g052670	0.059	AT2G46450	CNGC12	CYCLIC NUCLEOTIDE-GATED CHANNEL 12: Member of Cyclic nucleotide gated channel family. Positive regulator of resistance against avirulent fungal pathogen	1, 3
1	Top 14	Bo3g021540	0.059	AT1G51650		ATP synthase epsilon chain, mitochondrial*	2, 3
1	Top 15	Bo3g052480	0.059	AT2G02930	GSTF3	GLUTATHIONE S-TRANSFERASE F3: Encodes glutathione transferase belonging to the *phi* class of GSTs	1, 2, 3

1	Bot 1	Bo2g160770	-0.023	AT5G23940	PEL3	PERMEABLE LEAVES3 :A putative acyl-transferase. Mutation in this locus results in altered trichome phenotype. Additional phenotype includes altered cuticle layer	1, 3, 4
1	Bot 2	Bo00722s120	-0.022	AT1G72290	ATWSCP	WATER-SOLUBLE CHLOROPHYLL PROTEIN: Encodes a Kunitz-protease inhibitor, a water-soluble chlorophyll protein involved in herbivore resistance activation.	1, 2, 3
1	Bot 3	Bo6g098620	-0.022	AT1G67750		Probable pectate lyase 5*	1, 3
1	Bot 4	Bo6g112450	-0.022	AT1G70830	MLP28	MLP (= Major latex protein)-LIKE PROTEIN 28: Involved in cell wall polysaccaride metabolic process	1, 2, 3, 4
1	Bot 5	Bo9g008760	-0.020	AT3G29030	EXPA5	EXPANSIN A5: Involved in plant-type cell wall loosening	1, 3, 4
1	Bot 6	Bo1g051330	-0.019	AT4G28250	EXPB3	EXPANSIN B3: Putative beta-expansin/allergen protein, involved in plant-type cell wall loosening	1, 2, 3, 4
1	Bot 7	Bo9g177520	-0.019	AT5G04970	PMEI-PME47	Pectin methylesterase inhibitor; INVI-PMEI protein isoform which clustered with a PME	1, 3
1	Bot 8	Bo2g056170	-0.019	AT1G67750		Probable pectate lyase 5*	1, 3, 4
1	Bot 9	Bo7g117340	-0.019	AT4G35320		Uncharacterised protein*	1, 2, 3, 4
1	Bot 10	Bo01076s010	-0.019	AT2G32990	GH9B8	GLYCOSYL HYDROLASE 9B8: Enables cellulase activity; Endoglucanase 11*	1, 3
1	Bot 11	Bo02928s010	-0.018	AT1G62510	IPT1*	Encodes a putative adenylate isopentenyltransferase. It catalyzes the formation of isopentenyladenosine 5'-monophosphate (iPMP) from AMP and dimethylallylpyrophosphate (DMAPP), but it has a lower Km for ADP and likely works using ADP or ATP in plants. It is involved in cytokinin biosynthesis.	1, 3
1	Bot 12	Bo7g113100	-0.018	AT4G28410	RSA1	ROOT SYSTEM ARCHITECTURE 1: Tyrosine transaminase family protein	1, 2, 3
1	Bot 13	Bo8g115410	-0.018	AT1G04680		Probable pectate lyase 1*	1, 3, 4
1	Bot 14	Bo7g077650	-0.018	AT5G47500	PME5	PECTIN METHYLESTERASE 5: Predicted to encode a pectin methylesterase	1, 3, 4
1	Bot 15	Bo9g174870	-0.018	AT5G08000	PDCB2*/E13L3*	PLASMODESMATA CALLOSE-BINDING PROTEIN 2* / Glucan endo-1,3-beta-glucosidase-like protein 3: Encodes a member of the X8-GPI family of proteins. It localizes to the plasmodesmata and binds callose.	1, 3, 4

[1]Based on PLAZA v4.5: 1 = Best hit family (BLAST), 2 = Tree-based ortholog, 3 = Orthologous gene family, 4 = Anchor point (synteny)

Table S7: Top and bottom 15 loadings of PC2 of the *B. oleracea* "Kimmeridge" RNAseq dataset (Fig 4b). *Arabidopsis* orthologs of genes were identified with PLAZA 4.5 and their functional characterisation was done with TAIR and ENSEMBL databases. Genes with an asterisks were characterised based on protein structure with the UNIPROT database. Used abbreviations: BOL = *B. oleracea*, AT = *A. thaliana*.

PC	Rank	BOL gene	Loading score	AT ortholog (Best hit)	Name	Description/function	Orthology evidence
2	Top 1	Bo7g092700	0.060	AT5G49360	BXL1	BETA-XYLOSIDASE 1: Bifunctional beta-D-xylosidase/alpha-L-arabinofuranosidase required for pectic arabinan modification	1, 2, 3, 4
2	Top 2	Bo2g151530	0.058	AT5G49360	BXL1	BETA-XYLOSIDASE 1: Bifunctional *beta*-D-xylosidase/*alpha*-L-arabinofuranosidase required for pectic arabinan modification	1, 2, 3, 4
2	Top 3	Bo8g021770	0.052	AT1G44800	SIAR1	SILIQUES ARE RED 1: Functions as a bidirectional amino acid transporter that is crucial for the amino acid homeostasis of siliques. Member of nodulin MtN21-like transporter family	1, 3, 4
2	Top 4	Bo2g061060	0.050	AT1G68570	NPF3.1	NRT1(Nitrate Transporter)/ PTR(Peptide Transporter) FAMILY 3.1: A membrane localized GA transporter that is expressed in the root endodermis.	1, 3, 4
2	Top 5	Bo1g138550	0.046	AT3G13175	PRCE1	PSI-INTERACTING ROOT-CELL ENRICHED 1: Transmembrane protein	1, 2, 3, 4
2	Top 6	Bo6g003560	0.045	AT1G44800	SIAR1	SILIQUES ARE RED 1: Functions as a bidirectional amino acid transporter that is crucial for the amino acid homeostasis of siliques. Member of nodulin MtN21-like transporter family	1, 3, 4
2	Top 7	Bo3g061620	0.044	AT3G08040	FRD3	FERRIC REDUCTASE DEFECTIVE 3: Expressed in roots, but not shoots. Is likely to function in root xylem loading of an iron chelator or other factor necessary for efficient iron uptake out of the xylem or apoplastic space and into leaf cells.	1, 2, 3
2	Top 8	Bo3g064190	0.042	AT3G11480	BSMT1	Salicylate/benzoate carboxyl methyltransferase: The gene encodes a SABATH methyltransferase that methylates both salicylic acid and benzoic acid. It is highly expressed in flowers, induced by biotic and abiotic stress and thought to be involved in direct defence mechanism.	1, 3, 4
2	Top 9	Bo7g107470	0.041	AT4G21650	SBT3.13	SUBTILASE 3.13: Subtilisin-like protease*	1, 3

2	Top 10	Bo7g109980	0.041	AT4G25100	FSD1	FE SUPEROXIDE DISMUTASE 1: removal of superoxide radicals	1, 2, 3, 4
2	Top 11	Bo6g118620	0.039	AT1G74310	HSP101	HEAT SHOCK PROTEIN 101: Encodes ClpB1, which belongs to the Casein lytic proteinase/heat shock protein 100 (Clp/Hsp100) family. Involved in refolding of proteins which form aggregates under heat stress.	1, 2, 3, 4
2	Top 12	Bo7g114220	0.039	AT4G30110	HMA2	HEAVY METAL ATPASE 2: Encodes a protein similar to Zn-ATPase, a P1B-type ATPases transport zinc	1, 3, 4
2	Top 13	Bo6g116410	0.039	AT1G73010	PS2	PHOSPHATE STARVATION-INDUCED GENE 2: Encodes PPsPase1, a pyrophosphate-specific phosphatase catalysing the specific cleavage of pyrophosphate	1, 2, 3, 4
2	Top 14	Bo3g130430	0.038	AT1G53540	HSP17.6C	17.6 kDa class I heat shock protein 3: Member of the class I small heat-shock protein (sHSP) family, which accounts for the majority of sHSPs in maturing seeds	1, 3
2	Top 15	Bo9g078540	0.036	N/A			N/A
2	Bot 1	Bo1g123340	-0.098	AT3G16660		Pollen Ole e 1 allergen and extensin family protein*	1, 3, 4
2	Bot 2	Bo2g159220	-0.082	AT5G24770	VSP2	VEGETATIVE STORAGE PROTEIN 2: Has acid phosphatase activity dependent on the presence of divalent cations (Mg2+, Co2+, Zn2+, Mn2+) and anti-insect activity. Induced in response to abscisic acid, jasmonic acid, salt, water deficiency and wounding	1, 2, 3, 4
2	Bot 3	Bo6g119200	-0.082	AT1G75050		Pathogenesis-related thaumatin superfamily protein*	1, 3
2	Bot 4	Bo8g030940	-0.069	AT5G33370	CUS2	CUTIN SYNTHASE2: GDSL-motif esterase/acyltransferase/lipase. Enzyme group with broad substrate specificity that may catalyse acyltransfer or hydrolase reactions with lipid and non-lipid substrates.	1, 3
2	Bot 5	Bo3g045130	-0.067	N/A		Uncharacterised protein*	N/A
2	Bot 6	Bo1g016130	-0.065	AT4G29700	TIR1*	TRANSPORT INHIBITOR RESPONSE 1: Auxin receptor that mediates Aux/IAA proteins proteasomal degradation and auxin-regulated transcription.	1, 2, 3,
2	Bot 7	Bo3g055940	-0.063	N/A		Uncharacterised protein*	N/A

2	Bot 8	Bo5g136640	-0.060	AT3G11340	UGT76B1	UDP-DEPENDENT GLYCOSYLTRANSFERASE 76B1: Encodes a uridine diphosphate-dependent glucosyltransferase that conjugates isoleucic acid and modulates plant defence via glucosylation of N-hydroxypipecolic acid.	1, 3, 4
2	Bot 9	Bo3g087210	-0.059	AT2G13810	ALD1	AGD2-LIKE DEFENSE RESPONSE PROTEIN 1: ALD1 is a L-lysine alpha-aminotransferase. It is part of the pipecolic acid biosynthetic pathway, where it catalyses the biochemical conversion of lysine to epsilon-amino-alpha-ketocaproic acid (KAC) which is subject to subsequent transamination, cyclisation and isomerisation to form 2,3-dehydropipecolic acid	1, 2, 3
2	Bot 10	Bo5g027290	-0.056	AT1G19250	FMO1	FLAVIN-DEPENDENT MONOOXYGENASE 1: FMO1 is required for full expression of TIR-NB-LRR conditioned resistance to avirulent pathogens and for basal resistance to invasive virulent pathogens. Functions in an EDS1-regulated but SA-independent mechanism that promotes resistance and cell death at pathogen infection sites. FMO1 functions as a pipecolate N-hydroxylase and catalyses the biochemical conversion of pipecolic acid to N-hydroxypipecolic acid (NHP). NHP systemically accumulates in the plant foliage and induces systemic acquired resistance to pathogen infection.	1, 2, 3, 4
2	Bot 11	Bo2g075590	-0.056	AT1G72250	MDKIN1	MALECTIN DOMAIN KINESIN 1	1, 3
2	Bot 12	Bo8g106910	-0.053	AT1G14250	APY5*	Probable apyrase 5; Catalyses the hydrolysis of phosphoanhydride bonds of nucleoside tri- and di-phosphates*	1, 2, 3
2	Bot 13	Bo01002s040	-0.053	AT5G22570	WRKY38	WRKY DNA-BINDING PROTEIN 38: member of WRKY Transcription Factor; Group III	1, 2, 3
2	Bot 14	Bo4g192790	-0.053	AT2G42840	PDF1	PROTODERMAL FACTOR 1: Encodes a putative extracellular proline-rich protein is exclusively expressed in the L1 layer of vegetative, inflorescence and floral meristems and the protoderm of organ primordia.	1, 2, 3, 4
2	Bot 15	Bo3g052490	-0.050	AT4G02520	GSTF2	LUTATHIONE S-TRANSFERASE PHI 2: Encodes glutathione transferase belonging to the phi class of GSTs. The expression of this gene is upregulated by herbicide safeners such as benoxacor and fenclorim.	1, 2, 3

[1]Based on PLAZA v4.5: 1 = Best hit family (BLAST), 2 = Tree-based ortholog, 3 = Orthologous gene family, 4 = Anchor point (synteny)

Table S8: Differentially expressed genes (DEGs) in *B. oleracea* "Kimmeridge" with log2FC > 1 for the Bb.par vs Bb comparison. *Arabidopsis* orthologs of genes were identified with PLAZA 4.5 and their functional characterisation was done with TAIR and ENSEMBL databases. Genes with asterisks were characterised based on protein structure with the UNIPROT database. Used abbreviations: BOL = *B. oleracea*, AT = *A. thaliana*. Log2FC = Log2 fold change. Padj = adjusted p value. Bb.par = *B. brassicae* parasitised by parasitoid *D. rapae.*

Subset	log2FC	Padj	BOL gene	AT ortholog	AT gene name	Description/function	Orthology evidence[1]
Bb-Bb.par	7.99	5.74E-04	Bo4g111370	AT3G57260	PR2	PATHOGENESIS-RELATED PROTEIN 2: *beta* 1,3-glucanase	1, 3
Bb-Bb.par	2.59	1.14E-27	Bo1g158940	N/A		Uncharacterised protein*	N/A
Bb-Bb.par	1.71	9.42E-06	Bo6g068100	AT3G54590	EXT2	EXTENSIN 2: Encodes a hydroxyproline-rich glycoprotein. The mRNA is cell-to-cell mobile	1, 2, 3
Bb-Bb.par	1.65	2.00E-34	Bo8g020470	AT4G27990	YLMG1-2	One of four Arabidopsis homologs of bacterial ymlg proteins.	1, 2, 3
Bb-Bb.par	1.58	1.00E-03	Bo8g108490	AT1G12010	ACO3	Encodes a protein that appears to have 1-amino-cyclopropane-1-carboxylic acid oxidase activity based on mutant analyses. The mRNA is cell-to-cell mobile.	1, 3, 4
Bb-Bb.par	1.52	1.51E-06	Bo5g034220	AT1G21310	EXT3	EXTENSIN 3: Encodes extensin 3, which is a structural component which strengthens the primary cell wall	1, 2, 3, 4
Bb-Bb.par	1.31	2.20E-11	Bo3g034060	AT2G39020	NATA2	Probable N-acetyltransferase NATA1-like: Although this locus shares considerable sequence similarity with the adjacent NATA1 gene (At2g39030), they appear to encode genes with different functions. NATA1 is involved in the production of N-delta-acetylornithine, but, overexpression of At2g39020 in tobacco does not lead to the formation of this defence compound. The mRNA is cell-to-cell mobile.	1, 2, 3, 4
Bb-Bb.par	1.21	1.23E-04	Bo9g055990	AT3G47680		No apical meristem-associated C-terminal domain-containing protein*	1, 3
Bb-Bb.par	1.20	1.77E-15	Bo8g082410	AT3G52960	PRXIIE	PEROXIREDOXIN-II-E: Plastid localised peroxiredoxin.	1, 2, 3, 4
Bb-Bb.par	1.18	4.52E-09	Bo4g019630	AT2G43150	EXT21	EXTENSIN 21: Proline-rich extensin-like family protein	1, 3
Bb-Bb.par	1.17	7.51E-08	Bo4g119830	AT3G28550	EXT16	EXTENSIN 16: Proline-rich extensin-like family protein	1, 3
Bb-Bb.par	1.15	7.13E-03	Bo3g009970	N/A		Gibberellin-regulated protein 14*	N/A

Bb-Bb.par	1.14	2.61E-06	Bo6g068210	AT3G54690	SETH3	Sugar isomerase (SIS) family protein; Catalyses the reversible aldol-ketol isomerisation between D-ribulose 5-phosphate (Ru5P) and D-arabinose 5-phosphate (A5P)*	1, 2, 3
Bb-Bb.par	1.05	1.06E-27	Bo3g022110	AT4G27320	PHOS34	Contains a universal stress protein domain. Protein is phosphorylated in response to Phytophthora infestans zoospores and xylanase.	1, 2, 3, 4
Bb-Bb.par	1.05	6.51E-31	Bo4g185900	AT2G36000	EMB3114	EMBRYO DEFECTIVE 3114: Encodes an mTERF protein localised in the chloroplast stroma.	1, 2, 3, 4
Bb-Bb.par	1.04	1.17E-07	Bo4g194100	N/A		Extensin domain-containing protein*	N/A
Bb-Bb.par	1.01	2.23E-04	Bo6g092820	AT1G72020		TonB-dependent heme receptor A	1, 2, 3, 4
Bb-Bb.par	-1.00	1.47E-08	Bo9g069400	AT5G47220	ERF2	ETHYLENE RESPONSIVE ELEMENT BINDING FACTOR 2: Encodes a member of the ERF (ethylene response factor) subfamily B-3 of ERF/AP2 transcription factor family (ATERF-2). The protein contains one AP2 domain. Functions as activator of GCC box?dependent transcription. Positive regulator of JA-responsive defence genes and resistance to *F. oxysporum* and enhances JA inhibition of root elongation.	1, 3, 4
Bb-Bb.par	-1.01	9.36E-04	Bo4g140500	AT5G40780	LHT1	LYSINE HISTIDINE TRANSPORTER 1: Encodes LHT1 , a high-affinity transporter for cellular amino acid uptake in both root epidermis and leaf mesophyll.	1, 3, 4
Bb-Bb.par	-1.03	2.52E-03	Bo7g077880	AT5G47740	MCA23.6*	Adenine nucleotide alpha hydrolases-like superfamily protein	1, 2, 3, 4
Bb-Bb.par	-1.03	1.06E-05	Bo9g130640	AT5G57040	GLXI-LIKE;11	GLYOXALASE I-LIKE;11: Vicinal oxygen chelate superfamily member. Responds to NaCl,drought and high light stress.	1, 2, 3, 4
Bb-Bb.par	-1.09	1.75E-04	Bo9g169190	N/A		Uncharacterised protein	N/A
Bb-Bb.par	-1.10	4.13E-04	Bo5g003130	AT1G02390	GPAT2	GLYCEROL-3-PHOSPHATE SN-2-ACYLTRANSFERASE 2: putative sn-glycerol-3-phosphate 2-O-acyltransferase	1, 2, 3, 4
Bb-Bb.par	-1.11	6.72E-04	Bo3g178420	AT4G14365	XBAT34	XB3 ORTHOLOG 4 IN ARABIDOPSIS THALIANA: No E3 ubiquitin-protein ligase activity observed when associated with the E2 enzyme UBC8 in vitro.1*	1, 2, 3

Bb-Bb.par	-1.19	1.24E-05	Bo9g073240	AT2G18660	PNP-A/EXLB3	PLANT NATRIURETIC PEPTIDE A/EXPANSIN-LIKE B3 PRECURSOR: Encodes PNP-A (Plant Natriuretic Peptide A). PNPs are a class of systemically mobile molecules distantly related to expansins; their biological role has remained elusive. PNP-A contains a signal peptide domain and is secreted into the extracellular space. Co-expression analyses using microarray data suggest that PNP-A may function as a component of plant defence response and SAR in particular, and could be classified as a newly identified PR protein. It is stress responsive and can enhance its own expression.	1, 2, 3
Bb-Bb.par	-1.22	6.70E-03	Bo9g068130	AT5G35810	MIK22.12*	Ankyrin repeat family protein	1, 2, 3

[1]Based on PLAZA v4.5: 1 = Best hit family (BLAST), 2 = Tree-based ortholog, 3 = Orthologous gene family, 4 = Anchor point (synteny)

Table S9: Differentially expressed genes (DEGs) in *B. oleracea* “Kimmeridge” with log2FC > 1 for the Mp.par vs Mp comparison. *Arabidopsis* orthologs of genes were identified with PLAZA 4.5 and their functional characterisation was done with TAIR and ENSEMBL databases. Genes with an asterisks were characterised based on protein structure with the UNIPROT database. Used abbreviations: BOL = *B. oleracea*, AT = *A. thaliana*. Mp.par = *M. persicae* parasitised by parasitoid *A. colemani.*

Subset	log2FC	Padj	BOL gene	AT ortholog (Best hit)	AT gene name	Description/function	Orthology evidence[1]
Mp-Mp.par	2.43	2.08E-05	Bo8g093170	N/A		Extensin_2 domain-containing protein*	N/A
Mp-Mp.par	1.51	9.73E-04	Bo9g005630	AT4G02290	GH9B13	GLYCOSYL HYDROLASE 9B13: Endoglucanase, Endohydrolysis of (1->4)-beta-D-glucosidic linkages in cellulose, lichenin and cereal beta-D-glucans*	1, 3, 4
Mp-Mp.par	1.47	5.81E-04	Bo2g161180	AT5G60890	MYB34	MYB DOMAIN PROTEIN 34: Transcription factor involved in tryptophan gene activation and in indole-3-acetic acid (IAA) and indolic glucosinolates (IG) biosynthesis. Acts as a direct transcriptional activator of both Trp synthesis genes and Trp secondary metabolism genes*	1, 3
Mp-Mp.par	1.05	7.58E-07	Bo6g068210	AT3G54690	SETH3	Sugar isomerase (SIS) family protein; Catalyses the reversible aldol-ketol isomerisation between D-ribulose 5-phosphate (Ru5P) and D-arabinose 5-phosphate (A5P)*	1, 2, 3, 4
Mp-Mp.par	-1.00	3.45E-04	Bo3g009320	AT5G13740	ZIF1	ZINC INDUCED FACILITATOR 1: A member of the Major Facilitator Superfamily (MFS) of membrane proteins which are found in all organisms and transport a wide range of small, organic molecules. Involved in a mechanism of Zn sequestration, possibly by transport of a Zn ligand or Zn-ligand complex into vacuoles. The mRNA is cell-to-cell mobile	1, 2, 3, 4
Mp-Mp.par	-1.02	4.22E-04	Bo3g044790	AT4G10500	DLO1	DMR6-LIKE OXYGENASE 1: Converts salicylic acid (SA) to both 2,3-dihydroxybenzoic acid (2,3-DHBA) and 2,5-DHBA in vitro but only 2,3-DHBA in vivo. Component of a negative feedback regulation system of SA levels during senescence. Regulates both onset and progression of leaf senescence*	1, 3

Mp-Mp.par	-1.06	2.25E-04	Bo9g175880	AT5G06730	PER54*	Peroxidase 54: Removal of H2O2, oxidation of toxic reductants, biosynthesis and degradation of lignin, suberisation, auxin catabolism, response to environmental stresses such as wounding, pathogen attack and oxidative stress. These functions might be dependent on each isozyme/isoform in each plant tissue*	1, 3
Mp-Mp.par	-1.07	3.56E-04	Bo8g107090	AT1G14040	PHO1;H3	Phosphate transporter PHO1 homolog 3: Encodes a PHO1 homologue that is upregulated in response to Zn deficiency and is involved in Pi homeostasis in response to Zn deficiency. The mRNA is cell-to-cell mobile.	1, 2, 3
Mp-Mp.par	-1.07	9.57E-03	Bo3g058570	AT3G04720	PR4/HEL	PATHOGENESIS-RELATED 4 / Hevein-like preproprotein: Encodes a protein similar to the antifungal chitin-binding protein hevein from rubber tree latex. mRNA levels increase in response to ethylene and turnip crinkle virus infection. The mRNA is cell-to-cell mobile.	1, 2, 3
Mp-Mp.par	-1.08	3.37E-03	Bo9g002260	AT4G00350	ATI2*	ATG8-interacting protein 2: May be involved in salt stress-induced vesicle-to-vacuole trafficking pathway. Through its interaction with ATG8F, may enable delivery of the vesicle bodies to the vacuole by an autophagic pathway (Probable). Plays a role in seed germination in response to exogenous abscisic acid (ABA) treatment*	1, 3, 4
Mp-Mp.par	-1.08	4.31E-03	Bo8g059370	AT1G10340		Ankyrin repeat family protein*	1, 3
Mp-Mp.par	-1.08	3.95E-03	Bo9g113950	AT4G31790		Diphthine methyl ester synthase*: S-adenosyl-L-methionine-dependent methyltransferase that catalyses four methylations of the modified target histidine residue in translation elongation factor 2 (EF-2), to form an intermediate called diphthine methyl ester. The four successive methylation reactions represent the second step of diphthamide biosynthesis*	1, 2, 3
Mp-Mp.par	-1.08	3.72E-05	Bo7g096770	AT5G24530	DMR6	DOWNY MILDEW RESISTANT 6: Converts salicylic acid (SA) to 2,3-dihydroxybenzoic acid (2,3-DHBA) (By similarity).<br>Suppressor of immunity. Regulates negatively defence associated genes expression (e.g. PR-1, PR-2, and PR-5)*	1, 3

Mp-Mp.par	-1.08	4.04E-03	Bo7g108400	AT4G23010	UTR2	UDP-GALACTOSE TRANSPORTER 2: Sugar transporter involved in the transport of UDP-galactose form the cytoplasm into the Golgi apparatus*	1, 2, 3, 4
Mp-Mp.par	-1.11	2.79E-03	Bo6g077670	AT2G45920	PUB37*	U-box domain-containing protein 37: Functions as an E3 ubiquitin ligase*	1, 3
Mp-Mp.par	-1.11	2.25E-04	Bo1g010960	AT4G31800	WRKY18	WRKY DNA-BINDING PROTEIN 18: Pathogen-induced transcription factor. Binds W-box sequences in vitro. Forms protein complexes with itself and with WRKY40 and WRKY60. Constitutive expression of WRKY18 enhanced resistance to *P. syringae*, but its coexpression with WRKY40 or WRKY60 made plants more susceptible to both *P. syringae* and *B. cinerea*	1, 2, 3, 4
Mp-Mp.par	-1.11	3.67E-06	Bo3g036640	AT2G43570	CHI	Endochitinase: Random endo-hydrolysis of N-acetyl-beta-D-glucosaminide (1->4)-beta-linkages in chitin and chitodextrins*	1, 2, 3, 4
Mp-Mp.par	-1.11	2.97E-03	Bo3g052670	AT2G46450	CNGC12	CYCLIC NUCLEOTIDE-GATED CHANNEL 12: Member of Cyclic nucleotide gated channel family. Positive regulator of resistance against avirulent fungal pathogen.	1, 3
Mp-Mp.par	-1.12	1.64E-03	Bo4g111320	AT3G57260	PR2	PATHOGENESIS-RELATED PROTEIN 2: *beta* 1,3-glucanase	1, 3
Mp-Mp.par	-1.15	9.68E-06	Bo7g096760	AT5G24530	DMR6	DOWNY MILDEW RESISTANT 6: Converts salicylic acid (SA) to 2,3-dihydroxybenzoic acid (2,3-DHBA) (By similarity).<br>Suppressor of immunity. Regulates negatively defence associated genes expression (e.g. PR-1, PR-2, and PR-5)*	1, 3, 4
Mp-Mp.par	-1.16	3.41E-04	Bo3g052490	AT4G02520	GSTF2	GLUTATHIONE S-TRANSFERASE PHI 2: encodes glutathione transferase belonging to the phi class of GSTs.	1, 2, 3
Mp-Mp.par	-1.16	1.04E-03	Bo5g042840	AT1G26420	FOX5*	Berberine bridge enzyme-like 7*: Probable flavin-dependent oxidoreductase*	1, 3
Mp-Mp.par	-1.18	2.57E-03	Bo9g032500	N/A		Secreted protein*	N/A

Mp-Mp.par	-1.21	2.70E-04	Bo3g036290	AT2G42940	AHL16	AT-HOOK MOTIF NUCLEAR-LOCALISED PROTEIN 16: Encodes a nuclear matrix protein with AT-hook DNA binding motifs that acts in the maintenance of genomic integrity by silencing TEs and repeat-containing genes through epigenetic machinery. It interacts with FVE and MSI5 which are components of HDAC corepressor complexes.	1, 2, 4
Mp-Mp.par	-1.23	2.62E-03	Bo03358s010	AT2G36950	ATHMP20	HEAVY METAL ASSOCIATED PROTEIN 20: Heavy-metal-binding protein (By similarity). Involved in disease resistance*	1, 2, 3
Mp-Mp.par	-1.25	2.07E-03	Bo3g084270	N/A		Uncharacterised protein*	N/A
Mp-Mp.par	-1.25	3.16E-03	Bo3g021540	AT1G51650		ATP synthase subunit epsilon, mitochondrial*	2, 3
Mp-Mp.par	-1.26	1.64E-03	Bo2g013900	AT5G17760		AAA-ATPase*, P-loop containing nucleoside triphosphate hydrolases superfamily protein	1, 3
Mp-Mp.par	-1.26	7.92E-03	Bo1g078830	AT3G49340		Cysteine protease-like protein	1, 3, 4
Mp-Mp.par	-1.27	3.19E-06	Bo3g052480	AT2G02930	GSTF3	GLUTATHIONE S-TRANSFERASE F3: Encodes glutathione transferase belonging to the phi class of GSTs.	1, 2, 3
Mp-Mp.par	-1.32	9.12E-05	Bo6g119200	AT1G75050		Pathogenesis-related thaumatin superfamily protein*	1, 3
Mp-Mp.par	-1.35	8.54E-04	Bo9g174580	AT5G08260	scpl35	SERINE CARBOXYPEPTIDASE-LIKE 35	1, 3
Mp-Mp.par	-1.35	3.19E-06	Bo4g020920	AT2G43570	CHI	Endochitinase: Random endo-hydrolysis of N-acetyl-beta-D-glucosaminide (1->4)-beta-linkages in chitin and chitodextrins*	1, 2, 3, 4
Mp-Mp.par	-1.37	6.66E-05	Bo5g004490	AT1G04350		1-aminocyclopropane-1-carboxylate oxidase homolog 6: Encodes a protein whose sequence is similar to 2-oxoglutarate-dependent dioxygenase	1, 3, 4
Mp-Mp.par	-1.37	5.20E-03	Bo5g136640	AT3G11340	UGT76B1	UDP-DEPENDENT GLYCOSYLTRANSFERASE 76B1: Glycosylates the amino acid-related molecules isoleucic acid (2-hydroxy-3-methylpentanoic acid) and valic acid (2-hydroxy-3-methylbutyric acid). Acts as a negative regulator of salicylic acid (SA)-dependent plant defence in the absence of pathogens and promotes the jasmonate (JA) response*	1, 3, 4

Mp-Mp.par	-1.41	3.37E-03	Bo6g042330	AT1G51850	SIF2	STRESS INDUCED FACTOR 2: Malectin-like receptor-like kinase involved in MAMP mediated stomatal immunity. Interacts with BAK1/FLS2 signalling complex and subsequently phosphorylates and activates SLAC1.	1, 3
Mp-Mp.par	-1.43	3.19E-06	Bo01260s010	AT1G22990	HIPP22	HEAVY METAL ASSOCIATED ISOPRENYLATED PLANT PROTEIN 22: Heavy-metal-binding protein. Binds cadmium. May be involved in cadmium transport and play a role in cadmium detoxification*	1, 3
Mp-Mp.par	-1.46	4.33E-04	Bo8g071440	AT3G13950		Ankyrin*	1, 2, 3
Mp-Mp.par	-1.66	4.11E-03	Bo01002s040	AT5G22570	WRKY38	WRKY DNA-BINDING PROTEIN 38: member of WRKY Transcription Factor; Group III	1, 2, 3
Mp-Mp.par	-1.66	7.08E-06	Bo3g009330	AT5G13750	ZIFL1	ZINC INDUCED FACILITATOR-LIKE 1: Major facilitator superfamily (MFS) transporter probably involved in 2,4-dichlorophenoxyacetic acid (2,4-D) export*	1, 2, 3
Mp-Mp.par	-1.80	7.58E-07	Bo5g004500	AT1G04350		1-aminocyclopropane-1-carboxylate oxidase homolog 6: encodes a protein whose sequence is similar to 2-oxoglutarate-dependent dioxygenase	1, 3
Mp-Mp.par	-1.82	7.89E-04	Bo3g184370	AT1G53990	GLIP3	GDSL-MOTIF LIPASE 3: GDSL esterase/lipase 3*	1, 3
Mp-Mp.par	-1.95	4.78E-03	Bo4g031020	AT2G36950	ATHMP20	HEAVY METAL ASSOCIATED PROTEIN 20: Heavy-metal-binding protein (By similarity), Involved in disease resistance*	1, 2, 3, 4

[1]Based on PLAZA v4.5: 1 = Best hit family (BLAST), 2 = Tree-based ortholog, 3 = Orthologous gene family, 4 = Anchor point (synteny)

Table S10: Extended EPG Table. Both aphid species, where Bb represents *B. brassicae* and Mp represents *M. persicae,* were parasitised by their specialist parasitoid. Bb.par = *B. brassicae* parasitised by parasitoid *D. rapae.* Mp.par = *M. persicae* parasitised by its parasitoid *A. colemani*. C=Stylet cellular pathway, NP = non-probing, E1 = salivation in phloem, E2 = phloem ingestion, F = penetration difficulties in the cell wall, G = xylem ingestion, sig = significance symbol. Behaviours that were not performed by an aphid, were treated as zero values for mean, as missing values for the calculation of Mean, and as end of the recording for Latency.. Behaviours that were not performed on a plant line are annotated with NA', proportional variables (%) were tested with a χ2 test (only when represented by both performing and non-performing aphids), other variables were tested with Mann–Whitney U pairwise comparisons between parasitised and unparasitised aphids (*p < .05, **p < .01, ***p < .001).

		Brevicoryne brassicae							***Myzus persicae***						
**Behaviour**		**Bb**	**Bb.par**	**p value**	**sig**	**n Bb**	**n Bb.par**		**Mp**	**Mp.par**	**p value**	**sig**		**n Mp**	**n Mp.par**
**Pathway**	**Total duration of NP (min)**	25.2 ± 4.5	21.2 ± 4.3	0.2861		30	30		42.7 ± 4.8	34.3 ± 4.5	0.1727			30	30
	**Number of C events**	17.2 ± 1.6	17 ± 2.2	0.6196		30	30		28.1 ± 2.5	26.9 ± 2.4	0.7843			30	30
	**Total duration of C (min)**	277.1 ± 18.1	278.9 ± 19.5	0.9007		30	30		313.6 ± 18.5	310.4 ± 20.3	0.9941			30	30
	**Total number of C < 3 min**	2.2 ± 0.5	2.6 ± 1.3	0.2307		30	30		9.1 ± 1.4	8.2 ± 1.5	0.4588			30	30
**Salivation (E1)**	**Latency from 1st C to first E1 (min)**	183.2 ± 20.6	199.8 ± 26.1	0.6556		28	24		197.9 ± 29.8	152.8 ± 25.7	0.2788			26	26
	**Number of E1 events**	3.4 ± 0.4	3.3 ± 0.6	0.4464		30	30		6.3 ± 1	5.3 ± 1	0.3575			30	30
	**Total duration of E1 (min)**	17.2 ± 5.5	15.4 ± 2.8	0.7091		28	24		9.3 ± 1.4	12.6 ± 2	0.2870			26	26
	**Mean duration of E1 (min)**	6 ± 2.8	3.8 ± 0.4	0.6295		28	24		1.7 ± 0.4	2.9 ± 0.6	0.0093	**		26	26
	**Proportion of E1 in phloem phase (%)**	10.8 ± 2.7	10.6 ± 3.1	0.7527		29	24		13.3 ± 3.5	7.1 ± 1.6	0.2838			26	26
**Phloem (E2)**	**Latency from 1st C to first E2 (min)**	225.7 ± 28.2	245.2 ± 33.9	0.5882		23	17		220.8 ± 28.3	166.3 ± 34.1	0.1833			25	20
	**Aphids engaging in E2 (%)**	0.77	0.57	0.1709		30	30		0.83	0.67	0.2330			30	30

	**Total duration of E2 (min)**	121.3 ± 25.1	118.4 ± 30.1	1		23	17		92.5 ± 21.7	122.5 ± 28.5	0.3706			25	20
	**Mean duration of E2 (min)**	67.2 ± 17.6	46.4 ± 13.9	0.6262		23	17		67.2 ± 17.6	46.4 ± 13.9	0.3706			25	20
**Penetration Difficulties (F)**	**Aphids engaging in F (%)**	50%	47%	1		30	30		27%	13%	0.3329			30	30
	**Total duration of F (min)**	71.9 ± 7.8	73.8 ± 17.4	0.7800		15	14		107.7 ± 15.9	98.5 ± 33.2	0.9333			8	4
	**Mean duration of F (min)**	59.4 ± 7.5	60.5 ± 16.1	0.5045		15	14		94.4 ± 18.2	96 ± 35.2	0.8081			8	4
**Xylem drinking (G)**	**Aphids engaging in G (%)**	50%	80%	0.0304	*	30	30		33%	44%	0.7888			30	30
	**Total duration of G (min)**	42.5 ± 5.9	71.5 ± 13.9	0.1464		15	24		29.7 ± 4.5	68.7 ± 18.2	0.0358	*		10	12
	**Mean duration of G (min)**	28.9 ± 3.9	42.1 ± 8.1	0.7646		15	24		19.9 ± 2.5	46 ± 8.5	0.0056	**		10	12

Data represents the mean (± standard error)

Methods S1: Y-tube olfactometer bioassays and analysis

First, Kimmeridge plants were induced with 100 (parasitised) aphid adults for 96 hours or left uninduced for control plants (Fig 2ab). After induction, plant pots were wrapped in aluminium foil to exclude soil odours and two differently induced plants were placed separately in 30L glass jars (Fig 2c). A charcoal-filtered and humidified airflow (0.5 L min^{-1}) was led through the jars containing the plants with aphids into the Y-tube olfactometer. The Y-tube had a diameter of 0.9 cm, 5 cm arm length, 8.5 cm basis length and was tilted upward on a white wooden board with an angle of 40° towards a single light source (TL-D 58 W, Phillips, the Netherlands)[1].

After placing the plants in the glass jars, odours were allowed to flow for 15 min into the arms of the Y-tube olfactometer. At the start of each test, one mated *A. fuscicornis* female was released at the basis of the Y-tube and a choice was noted when the hyperparasitoid passed a drawn line at 1 cm from the end of either arm for 15 seconds. If 10 min had passed without the hyperparasitoid making a choice, a no-choice was recorded and excluded from statistical analysis. Each female was used only once, and each plant pair was subjected to choices of 8 or 10 hyperparasitoids (depending on hyperparasitoid availability). After half of the tests were completed, the airflows through the arms were swapped to correct for any unforeseen asymmetric bias of the setup. In total, 10-11 plant pairs were tested per treatment combination.

Choice data from the Y-tube assays (binary response: choice for one arm vs. the other) were analysed using generalized linear models (GLMs) with a binomial error distribution and logit link. We initially fitted generalized linear mixed models (GLMMs) with "Plant_pair" and "Day" included as a random intercept to account for multiple insects tested on the same plant pair. As the variance of this random effect was estimated as ~0, indicating no detectable plant-pair and day effects, the models were reduced to GLMs (excluding the random effects). Treatment effects were then evaluated with likelihood-ratio χ^2 tests (type II), and comparisons against H_0: 50:50 distribution (random choice) were performed on the logit scale using the emmeans package.

Methods S2: Collection and analysis of volatile organic compounds

Headspace collection of aphid-induced plants

To characterize the odour profiles of the aphid-infested plants, headspace samples were collected from 11 plants per treatment, resulting in a total of 55 samples. Headspace samples were collected in 30 L glass jars sealed with a rubber-lined glass lid containing an inlet for clean air. In our setup, each glass jar contained one infested plant or control plant (Fig 2d). A continuous inlet flow of clean, synthetic air (Air Synthetic 4.0 Monitoring from Linde Gas, Schiedam, The Netherlands) was used as a carrier of volatiles at a flow rate of 230 mL min^{-1}. Volatiles emitted by the plants were trapped by drawing air out of the glass jar at a suction rate of 200 mL min^{-1} through a stainless-steel tube filled with 200 mg Tenax TA (20/35 mesh; CAMSCO, Houston, TX, USA) for 2 h. Immediately after headspace collection, the Tenax TA cartridges were dry-purged under a stream of helium (50 mL min^{-1}) for 10 min at room temperature (21 ± 2 °C) to remove moisture before storage. To prevent any contribution from the collection set-up, the adsorbent material, and the analytical system, we routinely trapped volatiles from empty jars containing pots with soil wrapped in aluminium foil in the same way as the samples and included as background samples in subsequent analyses.

Separation and detection of volatile compounds

Desorption of volatiles from the Tenax TA, as well as separation and detection of volatiles, was carried out using a Thermal Desorber TD100-xr (Markes, Llantrisant, Glamorgan, UK) connected to a 7890B gas chromatography (GC) coupled to quadrupole-time-of-flight mass spectrometry (Q-ToF) both from Agilent (Agilent Technologies, USA). Released volatiles from the adsorbent at 250°C for 10 min under a helium flow of 30 ml min^{-1} were simultaneously re-collected in an electronically cooled sorbent trap (Markes) at 0°C. Once the desorption and recollection process was completed, volatile compounds were released from the cold trap by ballistic heating at 40°C s^{-1} to 280°C, which was then kept for 5 min, whilst the volatiles were transferred to a 30 mL x 0.25 mm ID x 1⍰m F.T. DB-5MS analytical column (Phenomenex, Torrance, CA, USA), placed inside the oven of a GC (Agilent Technologies) at a split ratio of 100:1 for further separation of plant volatiles. The GC oven temperature was initially held at 40°C for 2 min and was raised at 10°C min^{-1} to 100°C, held for 1 min and then raised at 5°C to

140°C and immediately was raised at 10°C to a final temperature of 280°C, where it was kept for 1 min under a constant helium flow of 1.2 ml min^{-1}.

Column effluents were ionized by electron impact ionization at 70 eV and detected with an accurate mass Q-TOF MS (Agilent Technologies), acquiring mass spectra from 35- 400 m/z at an acquisition rate of 5 spectra s^{-1}. The transfer line and ion source of the Q-TOF MS were set at 280 and 230°C, respectively. Chromatograms recorded for the presence of plant volatile compounds using MassHunter deconvolution software (Agilent Technologies, Inc 2008) were converted to Xcalibur data through a two-step rawdata-conversion program available in MetAlign software[3].

Automated baseline correction, peak selection (S/N > 3) and alignments of all extracted mass signals of the raw data were processed following an untargeted metabolomic workflow using MetAlign software, producing detailed information on the relative abundance of mass signals representing the available metabolites[3]. This is followed by reconstructing the extracted mass features into potential compounds using the MSClust software through data reduction employing unsupervised clustering and extraction of putative metabolite mass spectra[4]. Tentative identification of volatile metabolites was based on a comparison of the reconstructed mass spectra with those in the NIST 2014 and Wageningen Mass Spectral Database of Natural Products MS libraries, as well as experimentally obtained linear retention indices (LRIs).

Statistical analysis of volatile data

First, all obtained peak heights were divided by plant fresh biomass to correct for differences in plant size, this was used in subsequent analyses. Initially partial least squares-discriminant analysis (PLS-DA) as a tool to compare and correlate treatment groups. For this purpose, peak heights were mean scaled and centred. These models did not yield any predictive value (R2X = 0.421, R2Y = 0.0959, Q2 = -0.119). To identify differences in VOC blends of plants subjected to different treatments we then performed a permutational multivariate analysis of variance (PERMANOVA) with 1000 permutations. For this purpose, we used the adonis2 function of the *Vegan* package in R[2,6] on the Bray-Curtis distance matrix of the peak heights per compound of all samples. We used two models, of which the first model included treatment as sole factor. For the second model, the control was omitted from the dataset to include the factors "inducer identity", "parasitism rate" and their interaction factor.

To identify differences between the compositions of volatile blends, the median peak height values per treatment and compound were subjected to hierarchical clustering based on Euclidian distances with complete linkage as clustering criterion with the *pvClust* package[7]. Furthermore, p-values for hierarchical clustering were calculated with multiscale bootstrap resampling (10 000 bootstraps) based on approximately unbiased (AU) probability. Here, clusters with AU-probability larger than 95% are strongly supported by data. Following, the peak heights were scaled, centred and plotted in a heatmap based on compound class in the rows with the *ComplexHeatmap* package[8].

Differences in the compounds' peak heights between different treatments were statistically analysed with a one-way ANOVA followed by Tukey's HSD when the assumptions of normality (Shapiro-Wilkinson test) and equal variance of residuals (Bartlett's Test) were met. When violated, we subjected the data to a Kruskal-Wallis test followed by Dunn's test with a Benjamini-Hochberg correction. Both tests were carried out with a significance level of $\alpha<0.05$. Doing this, we used the packages *Dunn.test*[9], *Performance*[10] and *car*[11] using R version 4.2.0[2].

We identified several differentially emitted VOCs between treatments based on peak heights, using a DESeq2 approach (Love *et al.*, 2014). In short, we used the rounded peak height / plant biomass per compound, corrected for the cumulative peak height (SizeFactor) and calculated differential VOCs between treatments. We used a model with a combined factor for all treatments, which was relevelled to control treatment as baseline, and VOCs were classified as differential if they were different from the control with a false discovery rate lower than 0.05. To preserve true differences across conditions, we used apeGLM as shrinkage estimator[12]. For descriptive summaries we report DESeq2 size-factor–normalised peak heights (mean ± SE), which are the values underlying the DESeq2 model. Raw peak heights (per fresh weight; and uncorrected) can be found online on Figshare.

Because no calibrations were carried out using standard compounds, the analyses provide relative abundances of volatile compounds rather than absolute emission rates. We therefore did not compare summed signal intensities and focused on differences in the emission profiles and between individual compounds.

Methods S3: Transcriptomics of (parasitised) aphid-induced leaf samples

RNA isolation and sample preparation for RNA sequencing

Total RNA was extracted using the isolate-II RNA plant kit (Bioline Meridian, Germany), according to the manufacturer's instructions, with minor additions. We added a step incubated the samples for 15 minutes after adding cell lysis buffer to ground plant material, then the mixture was centrifuged for 10 minutes at 11,000 x *g*, and the supernatant was transferred in a clean tube. The final elution of RNA was performed in 50 µL nuclease-free water to the column. Sample concentration was measured with DeNoViX DS-11 spectrophotometer and quality with the Agilent 2100 bioanalyzer system and Agilent RNA 6000 nano kit. All samples had RIN > 8.5, whereas values higher than 7 are acceptable for RNA sequencing.

Quality control and read processing

DNBSEQ Eukaryotic mRNA library preparation, followed by 150 base pair paired-end RNA sequencing (DNB-seq) were performed at BGI Genomics, Hong Kong. Sequencing was performed to an average depth of 33.8 M reads (Table S7). After sequencing, the raw reads were filtered, including removal of adapter sequences, contamination, and low-quality reads. Quality of reads was assessed using FastQC and multiQC[13]. Reads were processed with Cutadapt[14] with settings: -m 75 -q 20 --no-indels. For indexing the genome (overhang = 74) and alignment of reads on the *B. oleracea* TO1000 genome V52[15], we used STAR[16] in quantMode to obtain read counts per gene. On average, 92.7% of the reads uniquely mapped on the genome (Table S7).

Differential gene expression analysis of selected contrasts

For contrasts between parasitised and unparasitised aphids of the same species (Fig. S4), we followed the approach of Karssemeijer et al. (2022) and analysed the relevant subsets separately. This involved re-estimating size factors, dispersions, and the apeglm prior within each subset, which can result in different results compared to the global model used for Fig. 5a.

SI references

1. Fatouros, N. E., Dicke, M., Mumm, R., Meiners, T. & Hilker, M. Foraging behavior of egg parasitoids exploiting chemical information. *Behav. Ecol.* **19**, 677–689 (2008).
2. R Core Team. A language and environment for statistical computing. (2024).
3. Lommen, A. MetAlign: Interface-driven, versatile metabolomics tool for hyphenated full-scan mass spectrometry data preprocessing. *Anal. Chem.* **81**, 3079–3086 (2009).
4. Tikunov, Y. M., Laptenok, S., Hall, R. D., Bovy, A. & de Vos, R. C. H. MSClust: a tool for unsupervised mass spectra extraction of chromatography-mass spectrometry ion-wise aligned data. *Metabolomics* **8**, 714–718 (2012).
5. Thévenot, E. A., Roux, A., Xu, Y., Ezan, E. & Junot, C. Analysis of the human adult urinary metabolome variations with age, body mass index, and gender by implementing a comprehensive workflow for univariate and OPLS statistical analyses. *J. Proteome Res.* **14**, 3322–3335 (2015).
6. Oksanen, J. *et al.* Vegan: community ecology package. (2015).
7. Suzuki, R. & Shimodaira, H. Pvclust: An R package for assessing the uncertainty in hierarchical clustering. *Bioinformatics* **22**, 1540–1542 (2006).
8. Gu, Z., Eils, R. & Schlesner, M. Complex heatmaps reveal patterns and correlations in multidimensional genomic data. *Bioinformatics* **32**, 2847–2849 (2016).
9. Dinno, A. dunn.test: Dunn’s test of multiple comparisons using rank sums. R package version 1.3.5 (2017).
10. Lüdecke, D., Ben-Shachar, M., Patil, I., Waggoner, P. & Makowski, D. Performance: An R package for assessment, comparison and testing of statistical models. *J. Open Source Softw.* **6**, 3139 (2021).
11. Fox, J. & Weisberg, S. *An {R} Companion to Applied Regression*. (Sage, Thousand Oaks, CA, 2019).
12. Zhu, A., Ibrahim, J. G. & Love, M. I. Heavy-Tailed prior distributions for sequence count data: Removing the noise and preserving large differences. *Bioinformatics* **35**, 2084–2092 (2019).
13. Andrews, S. FastQC: a quality control tool for high throughput sequence data. (2010).
14. Martin, M. Cutadapt removes adapter sequences from high-throughput sequencing reads. *EMBnet.journal* **17**, 10 (2011).
15. Parkin, I. A. P. *et al.* Transcriptome and methylome profiling reveals relics of genome dominance in the mesopolyploid *Brassica oleracea*. *Genome Biol.* **15**, 1–18 (2014).
16. Dobin, A. *et al.* STAR: Ultrafast universal RNA-seq aligner. *Bioinformatics* **29**, 15–21 (2013).
